# Multi-stage development process and model of steam chamber for SAGD production in a heavy oil reservoir with an interlayer

**DOI:** 10.1038/s41598-024-60747-7

**Published:** 2024-04-30

**Authors:** Ren-Shi Nie, Qingqiang Jiang, Yimin Wang, Jingcheng Liu, Jie Zhan, Letian Zhang, Yuanguang Li, Guotao Shen, Minghang Xu

**Affiliations:** 1 State Key Laboratory of Oil and Gas Reservoir Geology and Exploitation, Southwest Petroleum University, Chengdu, 610500 Sichuan China; 2 Exploration and Development Research Institute, Jianghan Oilfield Company, Sinopec, Wuhan, 430223 Hubei China; 3 Liquor Making Microbial Application and Detection Technology of Luzhou Key Laboratory, Luzhou Vocational and Technical College, Luzhou, 646000 China; 4 Institute of Petroleum and Gas Engineering, Chongqing University of Science and Technology, Chongqing, 401331 China; 5 School of Petroleum Engineering, Xi’an Shiyou University, Xi’an, 710065 China

**Keywords:** Heavy oil, SAGD, Horizontal well, Steam chamber, Interlayer, Model, Energy science and technology, Hydrology

## Abstract

Steam-assisted gravity drainage (SAGD) is an efficient thermal recovery technique for oil sands and extra heavy oil exploitation. The development of steam chamber goes through multi-stage physical processes for SAGD production in a heavy oil reservoir with an interlayer. In this study, considering the situation that an interlayer is located directly above a pair of horizontal wells, we analyzed the whole process of steam chamber development. We divided the whole process into stages I–V, which are the first rising stage, the first lateral expansion stage, the second rising stage, the second lateral expansion stage and the confinement stage, respectively. Particularly, we further divided stage II into 2 periods and stage IV into 3 periods. These stages and periods can help us understand the development process of steam chamber dominated by an interlayer more profoundly. Based on the divided stages and periods, we established different models of SAGD production by assuming different geometric shapes of steam chamber in different stages and periods. Oval shape was assumed in stages I and III, and inverse triangle shape was hypothesized in stages II, IV and V. The formulas of the front distance of steam chamber and the oil production rate of SAGD were deduced from the established models for different development stages. At the end, we performed two example applications to SAGD production in heavy oil reservoirs with an interlayer. The real oil production rates were matched very well with the theoretical oil production rates calculated by the deduced formulas, which implies the multi-stage development model of steam chamber is of reliability and utility.

## Introduction

**Figure 1:**
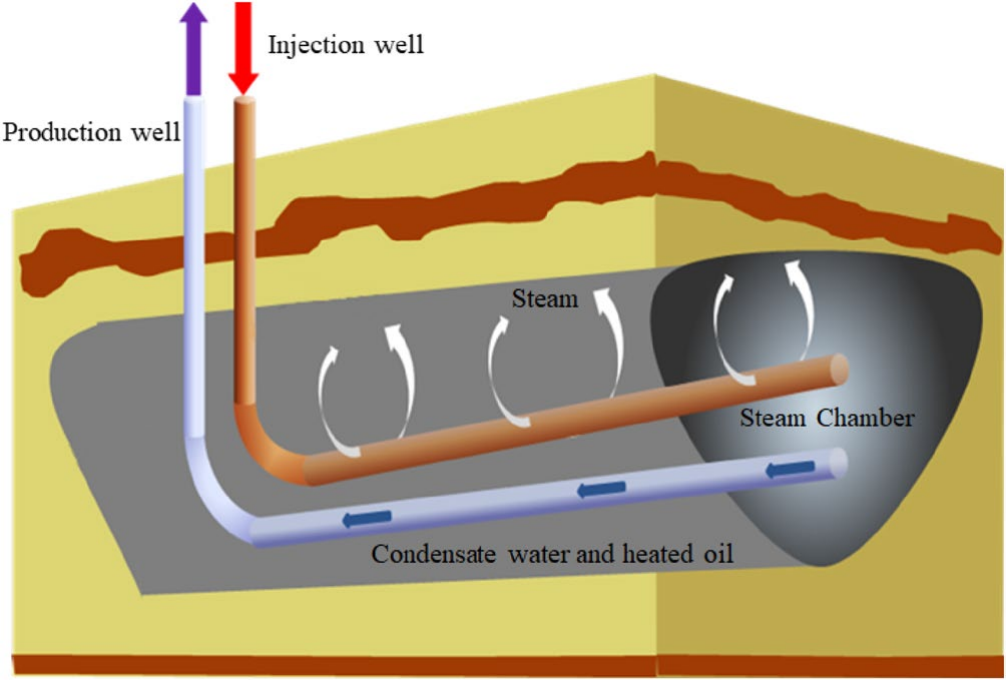
Diagram of SAGD.

According to the statistical review of global energy in 2021 from BP (British Petroleum) Company, the world's total oil-proved reserves at the end of 2020 were 1.732 trillion bbl, of which 40% was heavy oil^1,2^. Heavy oil resource has always played a significant role in meeting the world's energy needs. Steam-assisted gravity drainage (SAGD), originally proposed by Butler^3^, is the most efficient thermal recovery technique for Steam-assisted gravity drainage (SAGD) is an efficient thermal recovery technique for oil sands (API < 10) and extra heavy oil^4,5^, with high recovery rate, high oil production rate, and high gas-oil ratio^6^. In the SAGD process, a pair of parallel horizontal wells is drilled into an oil reservoir. Hot steam is injected into the reservoir through the upper well and rises until it reaches the cold formation. The steam releases its latent heat and condenses into water while the cold oil is heated. Condensate water and heated oil flow downward under the effect of gravity and the flooding effect of additional steam. After condensate water and heated oil flow downward, additional steam will occupy the space where condensate water and heated oil previously stay^7,8^. With the continuous injection of steam, a steam chamber is formed in the reservoir, as shown in Fig. 1.

The development of the steam chamber goes through three main stages: rising stage, lateral expansion stage and confinement stage (also named downward stage)^9–11^ At present, research on the development of steam chamber and oil production rate of SAGD mainly focuses on the lateral expansion stage, with less research on the rising stage and confinement stage^12^. Compared with the duration time of the lateral expansion stage, the duration time of rising stage is relatively shorter. Therefore, the rising process of steam was ignored in early researches^5,13^, which was not the case. Until 2012, Azad^14^ studied a circular steam chamber model for the rising stage and deduced a prediction formula of SAGD rate. Later, Nie et al.^15^ also researched a circular steam chamber model with consideration of various injection rates for the rising stage and obtained a prediction formula of SAGD rate. In addition, Zargar et al.^16,17^ established inverted triangle steam chamber models for the rising stage to predict the SAGD rate. In addition, Zhang et al.^18^ also established an inverted triangle model to predict the SAGD rate based on volume displacement theory. According to Darcy’s law and material balance theory, Guo et al.^5^ established a parabolic model in the rising stage and predicted the SAGD rate changing with time.

For the lateral expansion stage of steam chamber, Butler^19^ first proposed a SAGD oil drainage model, which assumed that the lateral interfaces of the steam chamber were a slope after the steam chamber reached the cap rock. This model considered that the location and dip angle of the slope interface was changed with the elapse of time and the bottom point of the slope interface was not fixed on the location of production well. The predicted SAGD rate calculated using the model was much higher than the real SAGD rate. In the same year, Butler and Stephens^3^ improved the model of Butler^15^ by fixing the bottom point of the slope interface on the location of production well and obtained a new SAGD rate formula. After that, Reis^20,21^ thought the Butler’s model was complex and inconvenient to use, so he made a simplification to the Butler’s model by assuming the shape of steam chamber as an inverted triangle in the lateral expansion stage. Later, on the basis of Reis’s model, many researchers investigated the issue of chamber development and SAGD rate in the lateral expansion stage through considering more actual factors, such as the changes of asphaltene and permeability with temperature^22,23^. In the last decades, more chamber shapes were introduced into the establishment of chamber model. Azad et al.^24^ thought the changes of steam chamber interface with the elapse of time looked like a group of linear geometry slices in the lateral expansion stage and established a slice model. Azad et al.^14^ adopted a circular interface model to describe the lateral expansion process of steam chamber. In addition, parabolic models were investigated to predict the SAGD rate and the lateral movement speed of steam chamber^5,8,25^. Moreover, Sabeti et al.^26^ adopted an exponential function to describe the interface shape of steam chamber to deduce a new formula of SAGD rate.

For the confinement stage of steam chamber, the research results available are relatively rare when compared with those of the lateral expansion stage. The confinement stage was also first proposed by Butler et al.^3^, but they did not establish a mathematical model for this stage. It was not until 2018 that Zargar et al.^16^ introduced the Butler’s inverted triangle concept in the lateral expansion stage into the model study of the confinement stage for the first time. Later, Zargar et al.^17^ applied their model to analyze the influence of constant steam injection rate on SAGD rate. Zhang et al.^18^ conducted a SAGD experiment and found the shape of steam chamber in the confinement stage could be approximately described using inverted triangle shape. Guo et al.^5^ adopted the parabola shape to model the interface of steam chamber in the confinement stage.

Some of the aforementioned literatures studied the continuity of steam chamber development through establishing comprehensive models. Two situations were researched: the rising and lateral expansion stages were synchronously modeled using the circular interface assumption of steam chamber^14^; and the whole process of the rising, lateral expansion and confinement stages were synchronously modeled using the inversely triangular interface assumption of steam chamber^17^ or the parabolic interface assumption^18^. In a word, only one interface shape was used to synchronously model multiple stages.

At present, all the analytical models available did not consider the influence of interlayers in reservoirs on the development of steam chamber. In the past, the impact of interlayers was mainly researched using numerical simulation methods^4,27–31^ and experimental approaches^32–36^. These research results show that interlayer has a great impact on the performance of SAGD. If a reservoir contains an interlayer, it is necessary to consider the influence of interlayer in the establishment of steam chamber model.

Therefore, the research objective of this paper is to establish a comprehensive model to simulate the performance of SAGD for a heavy oil reservoir with an interlayer. For real SAGD horizontal well production, the front position of steam chamber can be calculated using the established model and the development status of steam chambers in the strata with an interlayer can be known about. Here are some our innovation works: (1) interlayer is first introduced into the establishment of analytical model and the multi-stage development process of steam chamber controlled by an interlayer is demonstrated; (2) Mixed shape of steam chamber is first adopted to establish the comprehensive model; and this mixed shape is the assumption of oval shape in the rising stage and inversely triangular shape in lateral expansion and confinement stages; and (3) the effect of variable steam injection rate on SAGD production performance is analyzed.

The rest of the paper is structured as follows: "Physical model and development process of steam chamber" will introduce the physical model of SAGD production in a heavy oil reservoir with an interlayer and the entire development process of steam chamber controlled by the interlayer; "Mathematical model of SAGD production" will establish the mathematical models of SAGD production in different development stages of steam chamber and deduce the formulas of the front position of steam chamber and SAGD production rate; "Field application" perform field application, including the calculation of SAGD production rate, production history matching, the calculations of duration time and front position in different stages, etc.; and "Conclusions" will draw the research conclusions. The established model can be a good tool to calculate the front position of steam chamber and SAGD production rate.

## Physical model and development process of steam chamber

### Physical model

**Figure 2:**
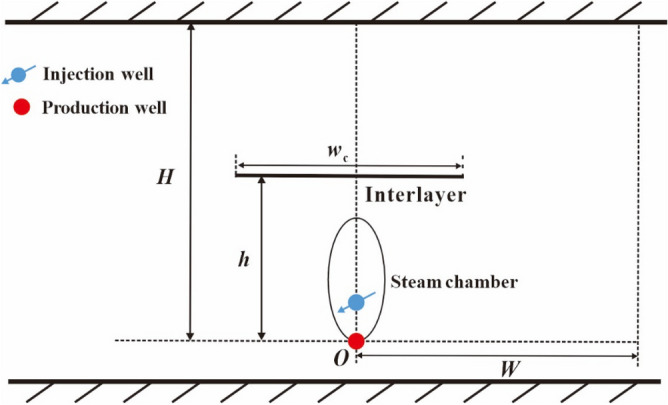
Profile diagram of SAGD production in a reservoir with an interlayer.

Figure 2 shows the diagram of SAGD production in a heavy oil reservoir with an interlayer. The top and bottom boundaries of the reservoir are considered as being impermeable and the interlayer is also considered as being impermeable. A pair of horizontal wells is drilled under the interlayer. The upper well is the injection well and the lower well is the production well. The distances of the interlayer and the reservoir top to the production well are denoted by *h* and *H*, respectively. The width of the interlayer is denoted by *w*_c_. The lateral drainage distance of SAGD is denoted by *W*, which is the distance from the lateral boundary of the reservoir to the horizontal well. After steam injection through the injection well, a steam chamber will be formed in the reservoir.

The basic assumptions of the physical model are as follow:

1. The reservoir properties, such as porosity, permeability and thermal diffusivity, are assumed as being constant;
2. The interior temperature of the steam chamber is uniformly distributed;
3. The reservoir temperature in the area un-swept by steam is assumed as a constant, which is equal to the initial reservoir temperature;
4. Heat loss is considered for the reservoir top and the interlayer;
5. The bottom point of the steam chamber (see point O in Fig. 2) is assumed to be always fixed on the position of the production well during the entire process of steam chamber development.

### Development process of steam chamber

For a heavy oil reservoir without an interlayer, the development process of steam chamber can be divided into 3 stages: the rising stage, the lateral expansion stage after steam reaches to the reservoir top and the confinement stage after steam reaches to the lateral drainage boundary. For a heavy oil reservoir with an impermeable interlayer, firstly, the steam chamber rises vertically; then, it expands laterally underneath the interlayer after encountering the impermeable interlayer; later, it rises again after bypassing the interlayer; after that, it expands laterally after encountering the impermeable top of the reservoir; and, finally, it expands downward after reaching to the lateral drainage boundary. Therefore, the development process of steam chamber can be divided into 5 stages for a reservoir with an interlayer: the first rising stage, the first lateral expansion stage, the second rising stage, the second lateral expansion stage and the confinement stage (downward stage).

The detailed descriptions about the 5 stages are as follows:

1. Stage I: the first rising stage

**Figure 3:**
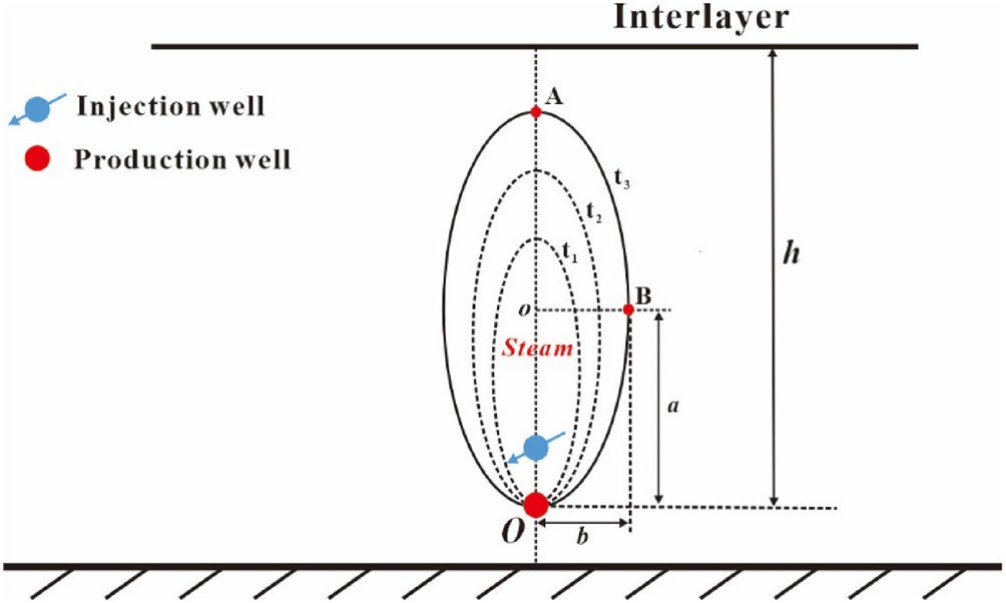
Diagram of steam chamber development in the first rising stage.

Steam chamber is formed in the reservoir after steam is injected through the injection well. The steam chamber gradually expands with the elapse of time in this stage. The interface of steam chamber is assumed as oval shape, as shown in Fig. 3. The major and minor radii of the oval chamber are denoted by *a* and *b*, respectively. The top vertex and co-vertex of oval chamber (see point A, B in Fig. 3) are called as the front position of steam chamber. The distance from the top vertex of oval chamber to the position of the production well is called as the perpendicular front distance of steam chamber in the first rising stage. The perpendicular front distance is just equal to the major diameter of oval chamber (2*a*). The distance from the co-vertex to the center of oval chamber is called as the lateral front distance of steam chamber in the first rising stage. The lateral front distance is just equal to the minor radius of oval chamber (*b*). The front distance can be used to quantitatively investigate the movement law of steam chamber. This stage ends when the top vertex of oval chamber reaches to the interlayer.

(2) Stage II: the first lateral expansion stage

**Figure 4:**
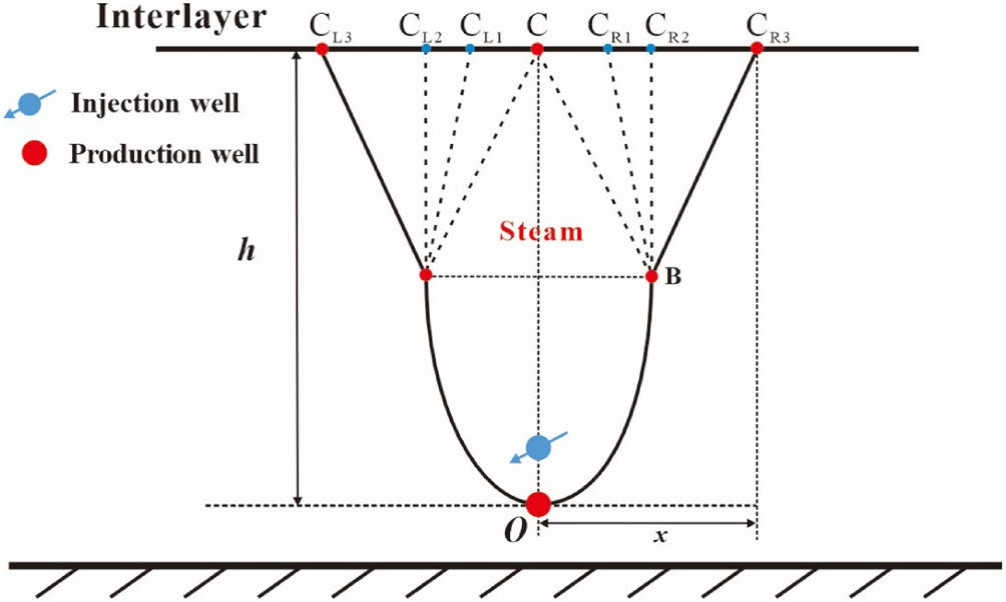
Diagram of steam chamber development in the early period of the first lateral expansion stage.

After the reaching of the top vertex of oval chamber to the interlayer (see point C in Fig. 4), the top vertex of the steam chamber becomes two top vertices, which move to the right and the left along the interlayer, respectively. We consider the same movement speed for the two top vertices. As shown in Fig. 4, the right top vertex gradually moves with the elapse of time from point C to point C_R1_, to point C_R2_ and to point C_R3_, and the left top vertex gradually moves with the elapse of time from point C to point C_L1_, to point C_L2_ and to point C_L3_. It is noted that the development of the steam chamber is bilaterally symmetric, so we will only describe the chamber development process to the right in the following analysis. The oval shape of the steam chamber gradually disappears. In this stage, the perpendicular front distance is equal to the distance from the interlayer to the production well (*h*) and the lateral front distance of steam chamber is equal to the lateral movement distance of the two top vertices (*x*). This lateral expansion stage can be divided into two periods: the early expansion period and the late expansion period.

i. The early period of the first lateral expansion stage.

In order to conveniently establish the development model of the steam chamber during the lateral expansion stage, we have to make a simplification to the interface shape of steam chamber. In the early period of this lateral expansion stage, the upper half part of steam chamber expands in the manner of approximate inverted triangle, which means that the co-vertex of oval chamber (point B) is deemed as a fixed end-point and points B, C and C_R3_ are the vertices of the inverted triangle, as shown in Fig. 4. In this period, the shape of the lower half part of steam chamber keeps unchanged.

ii. The late period of the first lateral expansion stage.

**Figure 5:**
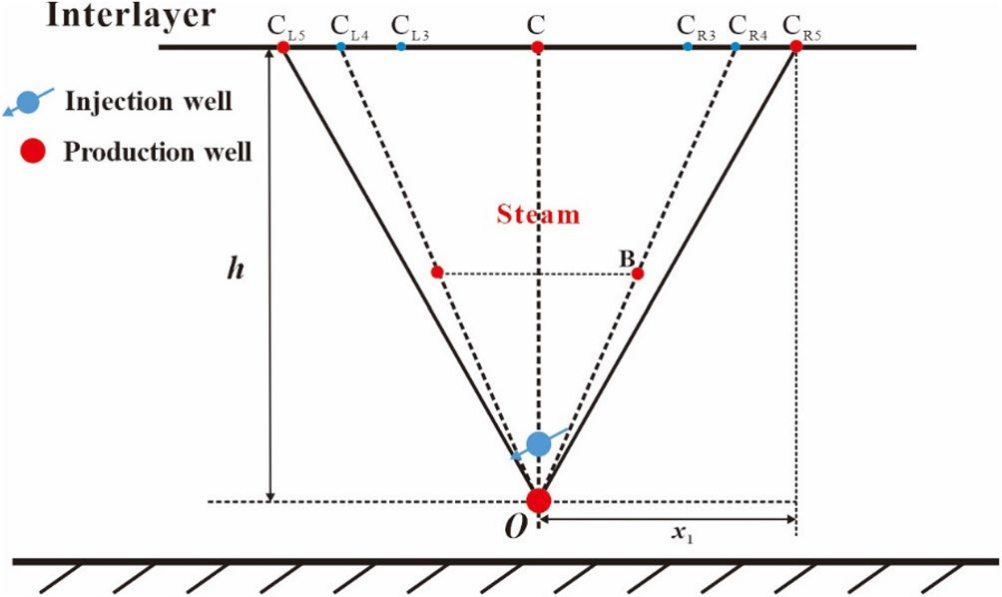
Diagram of steam chamber development in the late period of the first lateral expansion stage.

We connect point O and point B to form a line of OB, and then lengthen line OB to intersect with the interlayer and the intersection point is noted by point C_R4_. When the right top vertex moves to point C_R4_, the early period terminates and the late period begins. The two top vertices of the steam chamber continue to move to the right and the left along the interlayer, respectively. As shown in Fig. 5, the right top vertex gradually moves with the elapse of time from point C_R4_ to point C_R5_.

In this late period, the expansion of the steam chamber is still in the manner of approximate inverted triangle, which means that the position of the production well (point O) is deemed as a fixed end-point and points O, C_R5_ and C_L5_ are the vertices of the inverted triangle, as shown in Fig. 5. This assumption of the inverted triangle is also to conveniently establish the development model of the steam chamber.

(3) Stage III: the second rising stage

**Figure 6:**
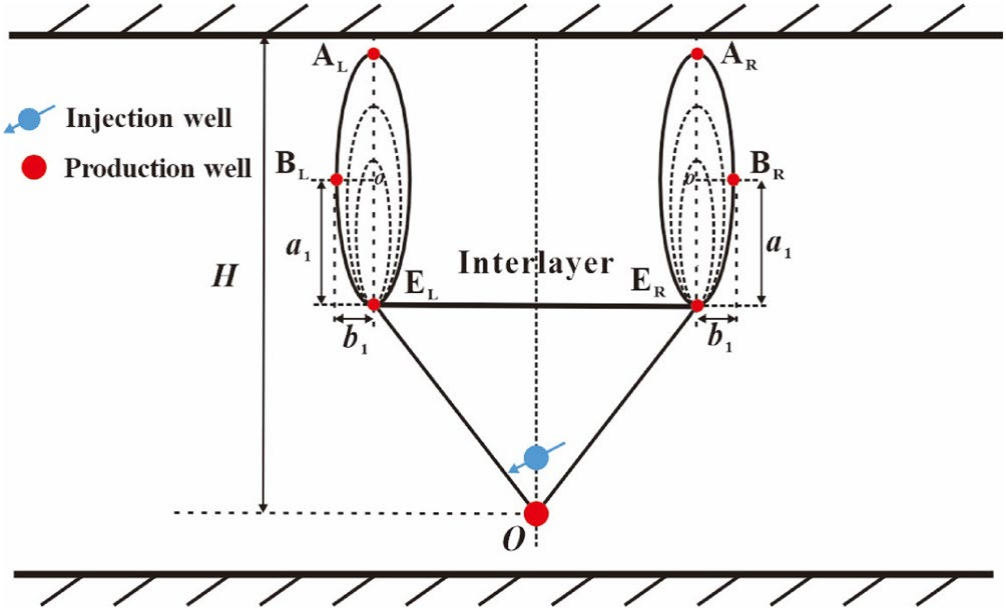
Diagram of steam chamber development in the second rising stage.

Figure 6 exhibits the development status of steam chamber in the second rising stage. We noted the interlayer edges by points E_R_ and E_L_, respectively, as shown in Fig. 6. When the right top vertex moves to point E_R_, the first lateral expansion stage terminates and the second rising stage begins. In this stage, the steam bypasses the interlayer, then rises at the same speed from points E_R_ and E_L_, respectively, and finally forms two sub-chambers. The steam chamber beneath the interlayer is a fixed inverted triangle with three vertices of points O, E_R_ and E_L_. Above the interlayer, the two steam sub-chambers expand in the manner of approximate oval. The two bottom vertices of oval sub-chambers are fixed on points E_R_ and E_L_, respectively. The two top vertices of oval sub-chambers are noted by points A_R_ and A_L_, respectively. The right co-vertex of the right sub-chamber is noted by point B_R_ and the left co-vertex of the left sub-chamber is noted by point B_L_. Points A_R_ and B_R_ represent the perpendicular and lateral front positions of the right sub-chamber, respectively. The perpendicular front distance is equal to the major diameter of oval sub-chamber (2*a*_1_). The lateral front distance is equal to the minor radius of oval sub-chamber (*b*_1_).

(4) Stage IV: the second lateral expansion stage

**Figure 7:**
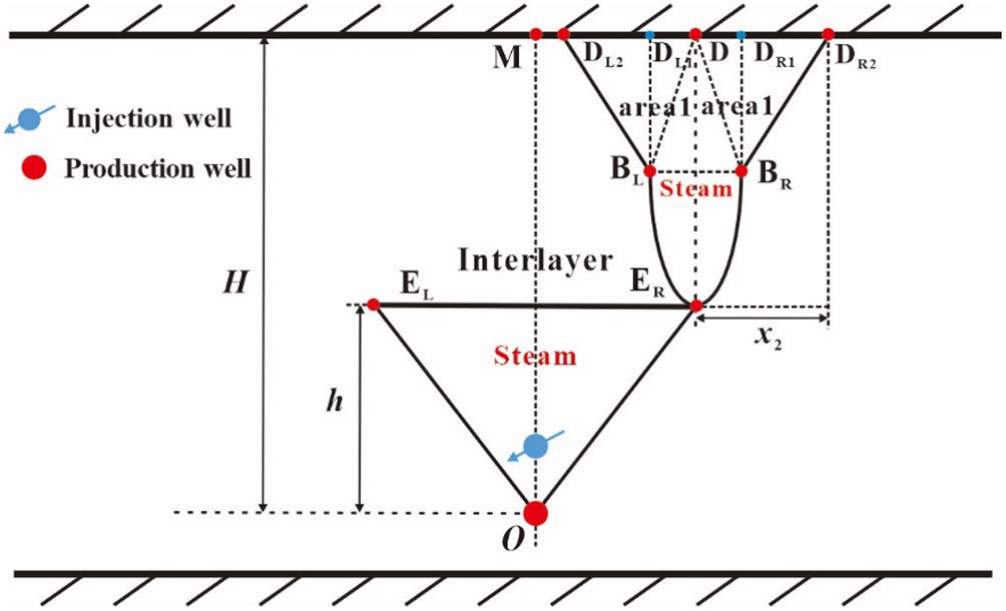
Diagram of the right sub-chamber development in the early lateral expansion period.

Because the two steam sub-chambers symmetrically develop, here we only describe the development process of the right sub-chamber. After the reaching of the top vertex of oval sub-chamber to the top boundary of the reservoir (see point D in Fig. 7), the top vertex of the steam sub-chamber becomes two top vertices, which move to the right and the left along the top boundary, respectively. As shown in Fig. 7, the right top vertex gradually moves with the elapse of time from point D to point D_R1_ and to point D_R2_, and the left top vertex gradually moves with the elapse of time from point D to point D_L1_ and to point D_L2_. The right sub-chamber can be divided into two areas: area 1 (the left area) and area 2 (the right area), as shown in Fig. 7.

(i) The development process of area 1 of the right sub-chamber.

This lateral expansion stage for area 1 of the right sub-chamber can be divided into 3 periods: the early lateral expansion period, the middle lateral expansion period and the late downward expansion period.

- The early lateral expansion period of area 1.

In the early lateral expansion period of area 1, the perpendicular front distance of the sub-chamber is equal to the distance from point E_R_ to point D (*H*–*h*) and the lateral front distance of the sub-chamber is equal to the lateral movement distance of the top vertex of the sub-chamber (*x*_2_). The upper half part of area 1 also expands in the manner of approximate inverted triangle, which means that the co-vertex of oval sub-chamber (point B_L_) is deemed as a fixed end-point and points B_L_, D and D_L2_ are the vertices of the inverted triangle, as shown in Fig. 7. In this period, the shape of the lower half part of area 1 keeps unchanged.

(b) The middle lateral expansion period of area 1.

**Figure 8:**
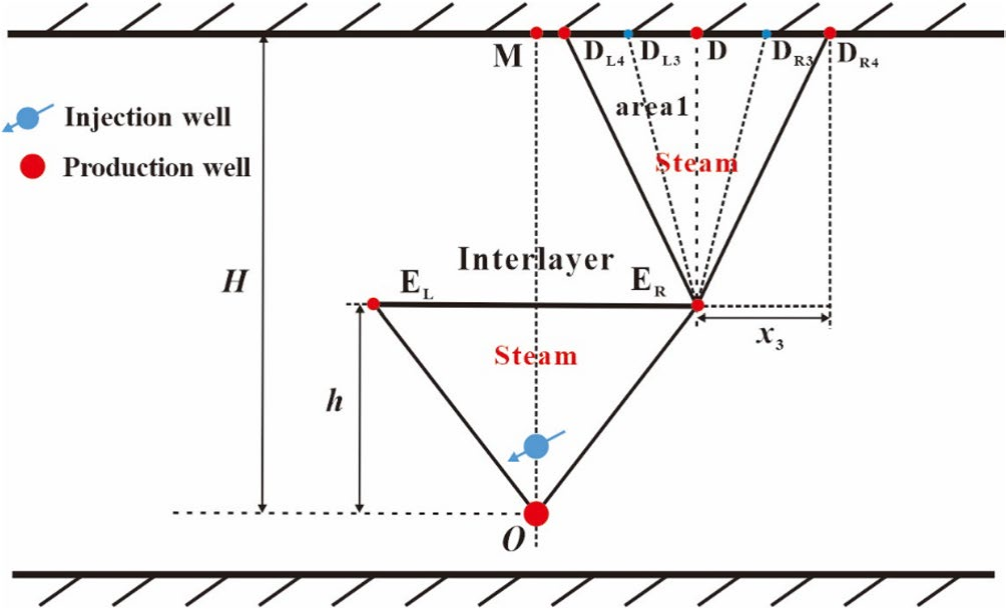
Diagram of the right sub-chamber development in the middle lateral expansion period.

In the middle lateral expansion period of area 1, the perpendicular and lateral front distances of the sub-chamber are equal to (*H*–*h*) and *x*_3_, respectively. Area 1 also expands in the manner of approximate inverted triangle, which means that point E_R_ is deemed as a fixed end-point and points E_R_, D and D_L4_ are the vertices of the inverted triangle, as shown in Fig. 8. This period terminates when the left top vertex of the sub-chamber reaches to point M.

(c) The late downward expansion period of area 1.

**Figure 9:**
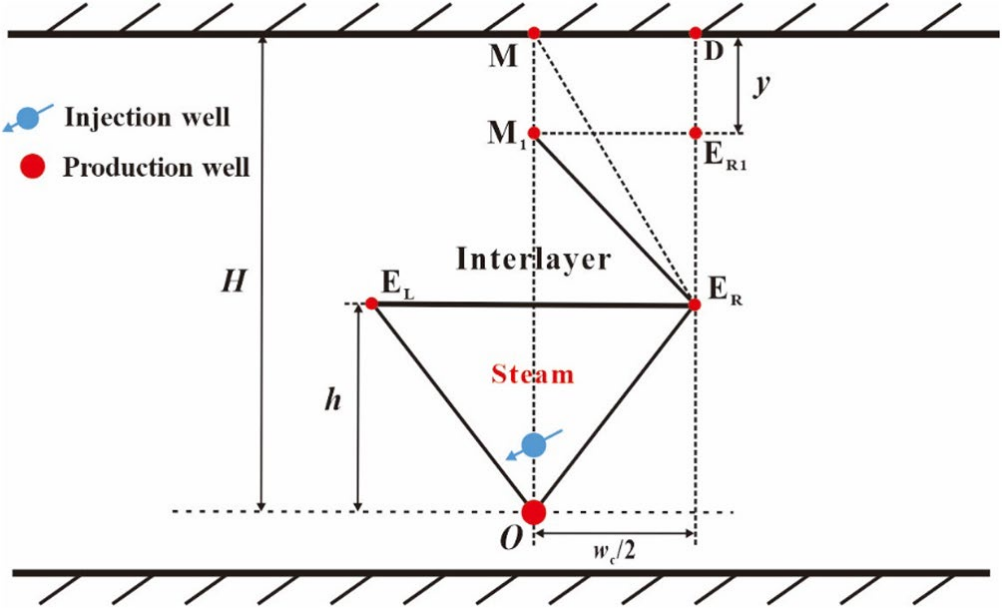
Diagram of area 1 in the late downward expansion period.

After the left top vertex of the sub-chamber reaches to point M, the late downward expansion period of area 1 begins. Area 1 becomes a trapezoid (DMM_1_E_R_) that is structured by a rectangle (DMM_1_E_R1_) and an inverted triangle (E_R_M_1_E_R1_), as shown in Fig. 9. In the development process of area 1 of the sub-chamber, the left top vertex of the inverted triangle moves downward from point M to M_1_, and the perpendicular movement distance is noted by *y*. This period terminates when the left top vertex of the inverted triangle reaches to the interlayer, and area 1 of the sub-chamber above the interlayer develops completely.

(ii) The development process of area 2 of the right sub-chamber.

This lateral expansion stage for area 2 of the right sub-chamber can be also divided into 3 periods: the early lateral expansion period, the middle lateral expansion period and the late lateral expansion period. It is noted that the development process of area 2 is the same as that of area 1 and area 2 is symmetric to area 1 in the early and middle lateral expansion periods, as shown in Figs. 7 and 8. When the middle lateral expansion period of area 1 terminates, area 2 still develops on the basis of the fixed end-point E_R_ and the right top vertex of area 2 reaches to point D_M_. The distance of line DD_M_ is equal to that of line DM.

**Figure 10:**
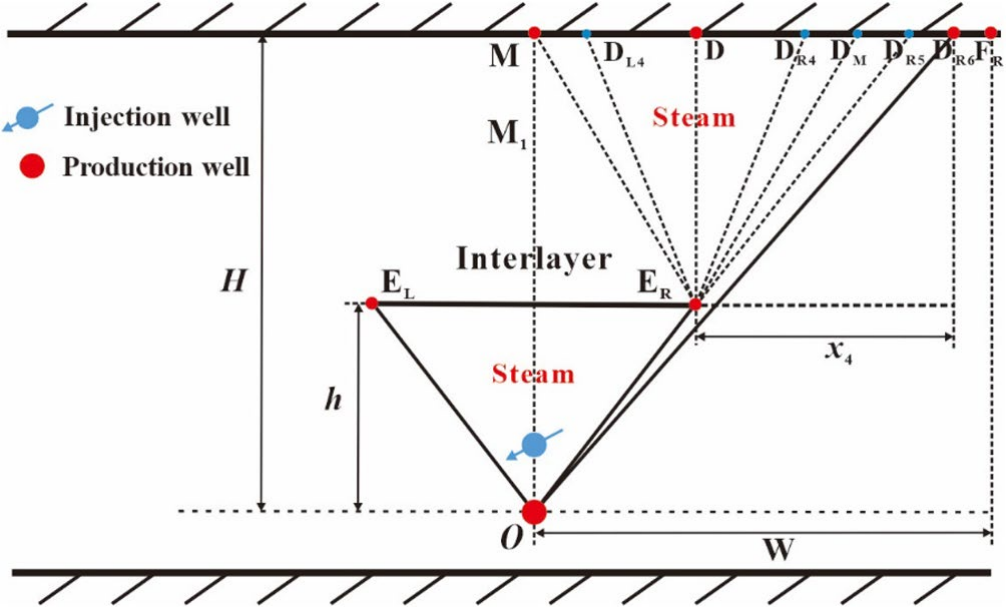
Diagram of area 2 in the late lateral expansion period.

We connect point O and point E_R_ to form a line of OE_R_, and then lengthen line OE_R_ to intersect with the reservoir top boundary and the intersection point is noted by point D_R5_. When the right top vertex of area 2 moves to point D_R5_, the middle lateral expansion period of area 2 terminates and the late lateral expansion period of area 2 begins. The right top vertex of area 2 continues to move to the right along the top boundary. As shown in Fig. 10, the right top vertex of area 2 gradually moves with the elapse of time from point D_R5_ to point D_R6_. The late lateral expansion period of area 2 terminates when the right top vertex of area 2 moves to the position of lateral drainage boundary of the reservoir (see point F_R_ in Fig. 10).

(5) Stage IV: the confinement stage

**Figure 11:**
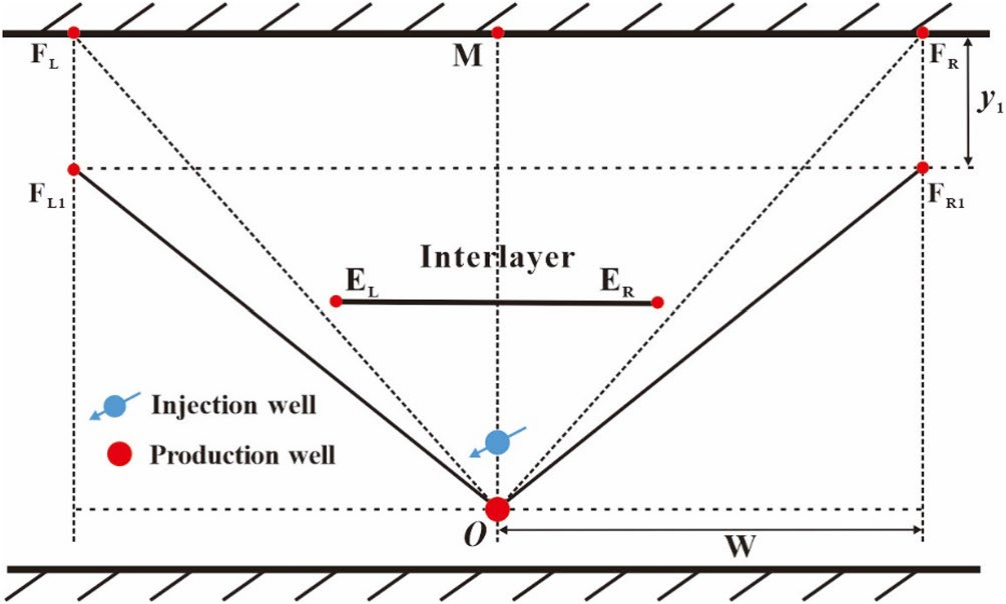
Diagram of steam chamber development in the confinement stage.

After the steam chamber reaches the lateral drainage edge (see point *F*_R_ in Fig. 10), the steam chamber has to grow downward because the growth of steam chamber is limited by the cap rock. The downward development stage of steam chamber is called as the confinement stage^3,36^. The steam chamber is structured by a rectangle (F_R_F_R1_F_L_F_L1_) and an inverted triangle (OF_R1_F_L1_), as shown in Fig. 11. In the development process of the steam chamber, the right top vertex of the inverted triangle moves downward from point F_R_ to F_R1_, and the perpendicular movement distance is noted by *y*_1_. The left top vertex of the inverted triangle does the same way. This stage terminates when steam is full of the whole drainage area of SAGD in the heavy oil reservoir.

**Figure 12:**
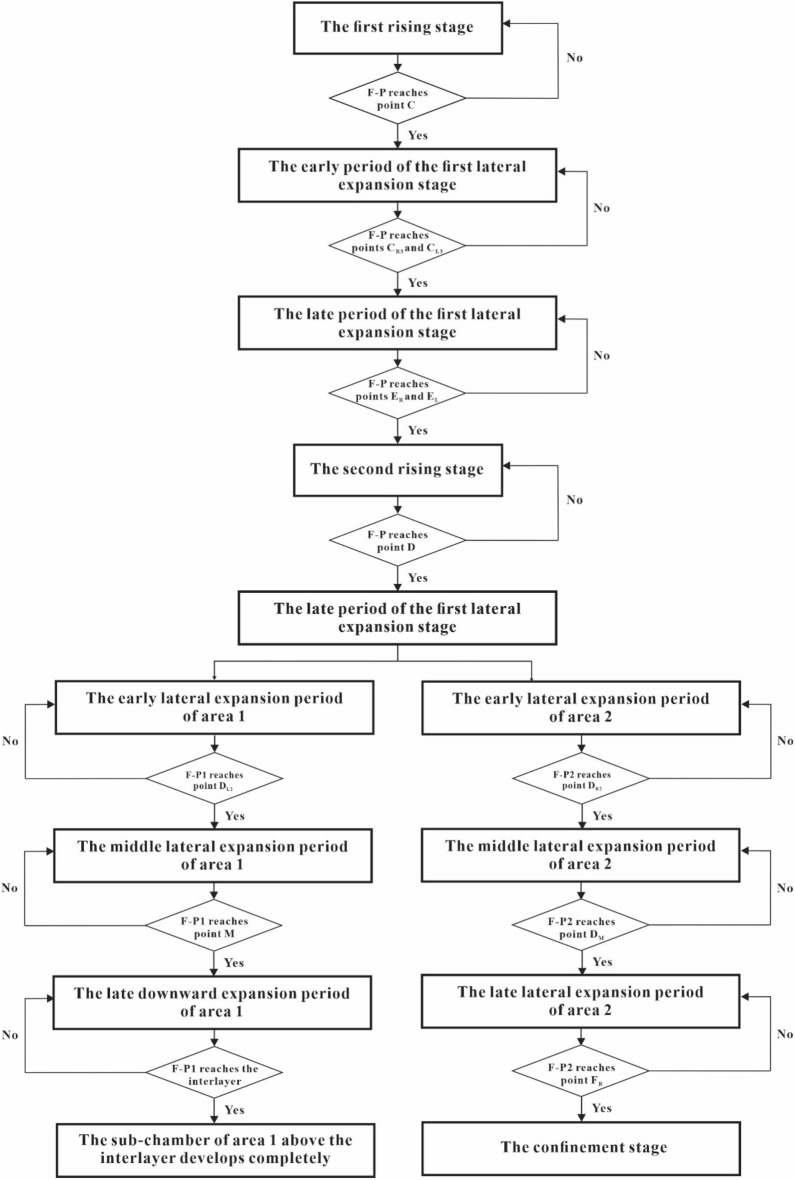
Flow chart of steam chamber development. “F-P” represents the front position of the steam chamber, and “F-P1” and “F-P2”represent the front positions of the sub-chambers of area 1 and area 2, respectively.

Figure 12 is a flow chart of steam chamber development, which can help us better understand the development process of steam chamber from the first rising stage to the confinement stage.

## Mathematical model of SAGD production

To conveniently describe the different stages of steam chamber development for the establishment of mathematical model, we denote *t*_C_ as the time of steam chamber reaching to point C, *t*_CR4_ as the time of steam chamber reaching to point C_R4_, *t*_ER_ as the time of steam chamber reaching to point E_R_, *t*_D_ as the time of steam chamber reaching to point D, *t*_DL3_ as the time of steam chamber reaching to point D_L3_, *t*_M_ as the time of steam chamber reaching to point M, *t*_ML_ as the time of steam chamber reaching to point M_L_, *t*_DR3_ as the time of steam chamber reaching to point D_R3_, *t*_DR5_ as the time of steam chamber reaching to point D_R5_, and *t*_FR_ as the time of steam chamber reaching to point F_R_. It is noted that *t*_C_ is the ending time of the first rising stage, *t*_CR4_ is the ending time of the early period of the first lateral expansion stage, *t*_ER_ is the ending time of the late period of the first lateral expansion stage, *t*_D_ is the ending time of the second rising stage, *t*_DL3_ is the ending time of the early lateral expansion period of area 1, *t*_M_ is the ending time of the middle lateral expansion period of area 1, *t*_ML_ is the ending time of the late downward expansion period of area 1, *t*_DR3_ is the ending time of the early lateral expansion period of area 2, *t*_DR5_ is the ending time of the middle lateral expansion period of area 2, and *t*_FR_ is the ending time of the late lateral expansion period of area 2.

### Mathematical model of the first rising stage

According to the Butler's theory, all mobile oil is drained away by steam and only residual oil is left in the steam chamber. Therefore, the oil production rate can be calculated by the chamber volume swept by steam.

Based on the material balance principle, the oil production rate per unit of horizontal well length before the steam chamber reaches the interlayer can be calculated by

1 $$q = 2{\uppi }\eta \rho_{{\text{o}}} \phi \Delta S_{{\text{o}}} a\frac{{{\text{d}}a}}{{{\text{d}}t}},{ (}t < t_{{\text{C}}} {)}$$

2 $$\Delta S_{{\text{o}}} = S_{{{\text{oi}}}} - S_{{{\text{or}}}}$$

where, *q* is oil production rate per unit of horizontal well length, kg/(m·d); *ρ*_o_ is oil density, kg/m^3^; *ϕ* is porosity, (j); *a* is major radius of oval chamber, m; *S*_oi_ is initial oil saturation, (j); *S*_or_ is residual oil saturation, (j); *t* is time, d; and *η* is experience coefficient, generally 0.7.

Steam is injected into the reservoir and heats the cold oil. The heated oil flows along the interface of steam chamber into the production well. The released heat rate per unit of horizontal well length can be calculated by

3 $$q_{{{\text{inject}}}} = \frac{{\chi q_{{\text{s}}} H_{{\text{s}}} }}{L}$$

where, *q*_inject_ is heat rate per unit of horizontal well length, J/(m day), *χ* is steam quality, (j); *q*_s_ is steam injection rate, kg/day; and *H*_s_ is latent heat of steam, J/kg; and *L* is horizontal well length, m.

To facilitate solving the model, we neglect the heat loss of the chamber interface and assume that the latent heat released by steam condensation heats the rock, oil and irreducible water. Based on the energy balance, the following equations are obtained:

4 $$q_{{{\text{inject}}}} = q_{{\text{r}}} + q_{{\text{o}}} + q_{{{\text{wc}}}}$$

5 $$q_{{\text{r}}} = \frac{{\rho_{{\text{r}}} }}{{\rho_{{\text{o}}} \phi \Delta S_{{\text{o}}} }}\left( {1 - \phi } \right)c_{{\text{r}}} \times \left( {T_{{\text{s}}} - T_{{\text{r}}} } \right)q$$

6 $$q_{{\text{o}}} = \frac{1}{{\Delta S_{{\text{o}}} }}S_{{{\text{oi}}}} c_{{\text{o}}} \times \left( {T_{{\text{s}}} - T_{{\text{r}}} } \right)q$$

7 $$q_{{{\text{wc}}}} = \frac{{\rho_{{\text{w}}} }}{{\rho_{{\text{o}}} \Delta S_{{\text{o}}} }}S_{{{\text{wc}}}} c_{{\text{w}}} \times \left( {T_{{\text{s}}} - T_{{\text{r}}} } \right)q$$

where *q*_r_ is heat absorption rate of rock per unit of horizontal well length, J/(m day); *q*_o_ is heat absorption rate of heavy oil per unit of horizontal well length, J/(m day); *q*_wc_ is heat absorption rate of irreducible water per unit of horizontal well length, J/(m day); *ρ*_r_ is rock density, kg/m^3^; *S*_wc_ is irreducible water saturation, (j); *ρ*_o_ is oil density, kg/m^3^; *ρ*_w_ is water density, kg/m^3^; *c*_r_ is specific heat of rock, J/(kg °C); *c*_o_ is specific heat of oil, J/(kg·°C); *c*_w_ is specific heat of water, J/(kg·°C); *T*_s_ is steam chamber temperature, °C; and *T*_r_ is initial reservoir temperature, °C.

Combine Eqs. (1) and (4)–(7):

8 $$\begin{aligned} \frac{{\chi q_{{\text{s}}} H_{{\text{s}}} }}{L} & { = }\left[ {\frac{{\rho_{{\text{r}}} }}{{\rho_{{\text{o}}} \phi \Delta S_{{\text{o}}} }}\left( {1 - \phi } \right)c_{{\text{r}}} + \frac{1}{{\Delta S_{{\text{o}}} }}S_{{{\text{oi}}}} c_{{\text{o}}} + \frac{{\rho_{{\text{w}}} }}{{\rho_{{\text{o}}} \Delta S_{{\text{o}}} }}S_{{{\text{wc}}}} c_{{\text{w}}} } \right] \\ & \times \left( {T_{{\text{s}}} - T_{{\text{r}}} } \right)2{\uppi }\rho_{{\text{o}}} \phi \Delta S_{{\text{o}}} \eta a\frac{{{\text{d}}a}}{{{\text{d}}t}},{ (}t < t_{{\text{c}}} {)} \\ \end{aligned}$$

Let,

$$A_{1} = \frac{{\chi q_{{\text{s}}} H_{{\text{s}}} }}{L}$$, $$B_{1} = 2{\uppi }\rho_{{\text{o}}} \left[ {\frac{{\rho_{{\text{r}}} }}{{\rho_{{\text{o}}} \phi \Delta S_{{\text{o}}} }}\left( {1 - \phi } \right)c_{{\text{r}}} + \frac{1}{{\Delta S_{{\text{o}}} }}S_{{{\text{oi}}}} c_{{\text{o}}} + \frac{{\rho_{{\text{w}}} }}{{\rho_{{\text{o}}} \Delta S_{{\text{o}}} }}S_{{{\text{wc}}}} c_{{\text{w}}} } \right] \times \left( {T_{{\text{s}}} - T_{{\text{r}}} } \right)\phi \Delta S_{{\text{o}}}$$.

Equation (8) is reduced to

9 $$A_{{1}} { = }B_{{1}} \eta a\frac{{{\text{d}}a}}{{{\text{d}}t}},{ (}t < t_{{\text{C}}} {)}$$

By integrating Eq. (9), the major radius of oval steam chamber is calculated by

10 $$a = \sqrt {\frac{{2\int_{0}^{t} {A_{1} {\text{d}}t} }}{{\eta B_{1} }}} ,{ (}t < t_{{\text{C}}} {)}$$

Then, the perpendicular front distance can be obtained by

11 $$h_{1} = 2a = 2\sqrt {\frac{{2\int_{0}^{t} {A_{1} {\text{d}}t} }}{{\eta B_{1} }}} ,{ (}t < t_{{\text{C}}} {)}$$

where, *h*_1_ is rising height of oval steam chamber, m.

Taking the derivative of Eq. (11) with respect to *t*, the rising speed of steam chamber can be gained by

12 $$v = \sqrt {\frac{{2A_{1}^{2} }}{{\eta B_{1} \int_{0}^{t} {A_{1} {\text{d}}t} }}} ,{ (}t < t_{{\text{C}}} {)}$$

where, *v* is rising speed of steam chamber, m/d.

By substituting Eq. (10) into Eq. (1), the oil production rate can be obtained by

13 $$q = \frac{{2{\uppi }\rho_{{\text{o}}} \phi \Delta S_{{\text{o}}} A_{1} L}}{{B_{1} }}$$

### Mathematic model of the first lateral expansion stage

1. The early period of the first lateral expansion stage.

After the steam chamber reaches the interlayer, the latent heat released from the condensation of steam is consumed for the expansion of steam chamber and heat loss.

Based on the energy balance principle, the latent heat rate per unit of horizontal well length can be expressed by

14 $$q_{{{\text{inject}}}} = q_{{{\text{in}}}} + q_{{{\text{loss}}}}$$

where, *q*_in_ is rate of heat absorption inside the steam chamber per unit horizontal well length, J/(m·d); and *q*_loss_ is rate of heat loss per unit horizontal well length, J/(m·d).

The consumed heat per unit horizontal well length of steam chamber expansion is calculated by

15 $$Q_{{{\text{in}}}} = \rho c(T_{{\text{s}}} - T_{{\text{r}}} )\frac{h}{2}{\text{d}}x$$

16 $$\rho c = (1 - \phi )\rho_{{\text{r}}} c_{{\text{r}}} + \phi (S_{{\text{o}}} \rho_{{\text{o}}} c_{{\text{o}}} + S_{{\text{w}}} \rho_{{\text{w}}} c_{{\text{w}}} )$$

where, *Q*_in_ is heat consumed per unit horizontal well length of steam chamber expansion, J/m; and *h* is distance from the interlayer to the production well.

Take the derivative of Eq. (15) with respect to *t*:

17 $$q_{{{\text{in}}}} = \frac{{{\text{d}}Q_{{{\text{in}}}} }}{{{\text{d}}t}} = \rho c(T_{{\text{s}}} - T_{{\text{r}}} )\frac{h}{2}\frac{{{\text{d}}x}}{{{\text{d}}t}},{ (}t_{{\text{C}}} { < }t{ < }t_{{{\text{CR4}}}} )$$

We consider that heat loss is consumed at both the interlayer and the side interface of steam chamber, so the heat loss can be divided into two parts:

18 $$q_{{{\text{loss}}}} = q_{{{\text{layer}}}} + q_{{{\text{side}}}}$$

where, *q*_layer_ is rate of heat loss at the interlayer per unit horizontal well length, J/(m day); and *q*_side_ is rate of heat loss at the side interface of steam chamber per unit horizontal well length; J/(m day).

Carslaw and Jaeger^37^ built and solved a heat loss model for impermeable cap rock to deduce the rate formula of heat loss after steam chamber reaches to cap rock:

19 $$q_{{{\text{cap}}}} = 2\int_{0}^{t} {(T_{{\text{s}}} - T_{{\text{r}}} )\sqrt {\frac{{\lambda_{{{\text{cap}}}} \rho_{{{\text{cap}}}} c_{{{\text{cap}}}} }}{{{\uppi }(t - \tau )}}} \frac{{{\text{d}}x}}{{{\text{d}}\tau }}{\text{d}}\tau }$$

where, *q*_cap_ is rate of heat loss at the cap rock per unit horizontal well length, J/(m^2^ day); *λ*_cap_ is thermal conduction coefficient of cap rock, J/(m day °C); *ρ*_cap_ is cap rock density, kg/m^3^; *c*_cap_ is cap rock heat capacity, J/(kg·°C); and *τ* is integral variable respect to time, day.

We introduce the rate formula of heat loss for cap rock into the calculation of heat loss for interlayer rock:

20 $$q_{{{\text{layer}}}} = 2\int_{{t_{{\text{C}}} }}^{t} {(T_{{\text{s}}} - T_{{\text{r}}} )\sqrt {\frac{{\lambda_{{{\text{layer}}}} \rho_{{{\text{layer}}}} c_{{{\text{layer}}}} }}{{{\uppi }(t - \tau )}}} \frac{{{\text{d}}x}}{{{\text{d}}\tau }}{\text{d}}\tau } ,{ (}t_{{\text{C}}} { < }t < t_{{{\text{CR4}}}} )$$

where, *λ*_layer_ is thermal conduction coefficient of the interlayer, J/(m day·°C); *ρ*_layer_ is interlayer density, kg/m^3^; *c*_layer_ is interlayer heat capacity, J/(kg·°C); and *t*_c_ is time for the front position to reach point C, day.

Through extensive calculations and comparison with STARS results, Shaolei et al.^8^ concluded that the heat loss around the steam chamber is 1/6 of the heat loss from the cap. We assume the same ratio relationship of heat loss between the side interface of steam chamber and the interlayer:

21 $$q_{{{\text{side}}}} = \frac{1}{6}q_{{{\text{layer}}}}$$

Combining Eqs. (3) and (14)−(21), we obtain

22 $$\frac{{\chi q_{{\text{s}}} H_{{\text{s}}} }}{L} = \frac{7}{3}\int_{{t_{{\text{C}}} }}^{t} {(T_{{\text{s}}} - T_{{\text{r}}} )\sqrt {\frac{{\lambda_{{{\text{layer}}}} \rho_{{{\text{layer}}}} c_{{{\text{layer}}}} }}{{{\uppi }(t - \tau )}}} } \frac{{{\text{d}}x}}{{{\text{d}}\tau }}{\text{d}}\tau + \rho c(T_{{\text{s}}} - T_{{\text{r}}} )\frac{h}{2}\frac{{{\text{d}}x}}{{{\text{d}}t}},{ (}t_{{\text{C}}} { < }t{ < }t_{{{\text{CR4}}}} )$$

By solving Eq. (22) (see Appendix A), we obtain the expression of the front distance of steam chamber:

23 $$x = \int_{{t_{{\text{C}}} }}^{t} {\frac{{A_{2} }}{C}{\text{e}}^{{[\frac{{B_{2} }}{C}\Gamma (0.5)]^{2} \tau }} {\text{erfc}}[\frac{{B_{2} }}{C}\Gamma (0.5)\sqrt \tau ]{\text{d}}\tau } ,{ (}t_{{\text{C}}} { < }t < t_{{{\text{CR4}}}} )$$

where, $A_{2} = \frac{{\chi q_{{\text{s}}} H_{{\text{s}}} }}{L}$; $B_{2} = \frac{7}{3}(T_{{\text{s}}} - T_{{\text{r}}} )\sqrt {\frac{{\lambda_{{{\text{layer}}}} \rho_{{{\text{layer}}}} c_{{{\text{layer}}}} }}{{\uppi }}}$; $C = \rho c(T_{{\text{s}}} - T_{{\text{r}}} )\frac{h}{2}$; and Г( ) is gamma function.

Based on the material balance principle, the oil production rate per unit of horizontal well length after the steam chamber reaches the interlayer can be calculated by

24 $$q = \frac{1}{2}\rho_{{\text{o}}} \phi \Delta S_{{\text{o}}} h\frac{{{\text{d}}x}}{{{\text{d}}t}},{ (}t_{{\text{C}}} { < }t{ < }t_{{{\text{CR4}}}} )$$

Substituting Eq. (23) into Eq. (24), we obtain

25 $$q = \frac{1}{2}\rho_{{\text{o}}} \phi \Delta S_{{\text{o}}} h\frac{{A_{2} }}{C}{\text{e}}^{{[\frac{{B_{2} }}{C}\Gamma (0.5)]^{2} (t - t_{{\text{C}}} )}} {\text{erfc}}[\frac{{B_{2} }}{C}\Gamma (0.5)\sqrt {t - t_{{\text{C}}} } ],{ (}t_{{\text{C}}} { < }t{ < }t_{{{\text{CR4}}}} )$$

where, erfc is error function.

(2) The late period of the first lateral expansion stage.

After the steam chamber moves to point C_R4_ (Fig. 5), the latent heat released by steam per unit horizontal well length during the late period of the first lateral expansion stage can be calculated by

26 $$Q_{{{\text{inject}}}} = \frac{{\chi H_{{\text{s}}} }}{L}\overline{q}_{{\text{s}}} (t - t_{{{\text{CR4}}}} ),{ (}t_{{{\text{CR4}}}} < t < t_{{{\text{ER}}}} )$$

27 $$\overline{q}_{{\text{s}}} = \frac{{\sum\limits_{{i = t_{{{\text{CR4}}}} }}^{t} {q_{{{\text{s,}}i}} } }}{{t - t_{{{\text{CR4}}}} }},{ (}t_{{{\text{CR4}}}} < t < t_{{{\text{ER}}}} )$$

where, *Q*_inject_ is latent heat released by steam per unit horizontal well length, J/m; *t*_CR4_ is time of steam chamber reaching to point C_R4_, day; $\overline{q}_{{\text{s}}}$ is average rate of steam injection from *t*_CR4_ to *t*, kg/day; *t*_ER_ is time of steam chamber reaching to point E_R_, day; and *q*_s,*i*_ is steam injection rate on the *i*th day, kg/day.

In the late period of the first lateral expansion stage, the heat consumed per unit horizontal well length of steam chamber expansion can be calculated by

28 $$Q_{{{\text{in}}}} = \rho c(T_{{\text{s}}} - T_{{\text{r}}} )h\overline{V} (t - t_{{{\text{CR4}}}} ),{ (}t_{{{\text{CR4}}}} < t < t_{{{\text{ER}}}} )$$

where, $\overline{V}$ is average movement speed of the steam chamber during the late period of the first lateral expansion stage, m/day.

Based on the energy balance principle, the latent heat per unit of horizontal well length can be expressed by

29 $$Q_{{{\text{inject}}}} = Q_{{{\text{in}}}} + Q_{{{\text{loss}}}}$$

30 $$Q_{{{\text{loss}}}} = \int_{{t_{{{\text{CR4}}}} }}^{t} {q_{{{\text{loss}}}} {\text{d}}t}$$

where, *Q*_loss_ is heat loss per unit horizontal well length of steam chamber expansion, J/m.

According to Eq. (19), the heat loss of the interlayer is expressed by

31 $$q_{{{\text{layer}}}} = 4(T_{{\text{s}}} - T_{{\text{r}}} )\sqrt {\frac{{\lambda_{{{\text{layer}}}} \rho_{{{\text{layer}}}} c_{{{\text{layer}}}} }}{{\uppi }}} [\overline{V}_{1} (\sqrt {t - t_{{\text{C}}} } - \sqrt {t - t_{{{\text{CR4}}}} } ) + \overline{V} \sqrt {t - t_{{{\text{CR4}}}} } ],{ (}t_{{{\text{CR4}}}} < t < t_{{{\text{ER}}}} )$$

32 $$\overline{V}_{1} = \frac{{x_{{CC_{R4} }} }}{{t_{{{\text{CR4}}}} - t_{{\text{C}}} }}$$

33 $$x_{{{\text{CCR4}}}} = \beta h$$

where, $\overline{V}_{{1}}$ is average movement speed of steam chamber from *t*_C_ to *t*_CR4_, m/d; and *x*_CCR4_ is length of line CC_R4_, m.

Combining Eqs. (21) and (26)–(33), we obtain

34 $$\begin{aligned} & \frac{{\chi \overline{q}_{{\text{s}}} H_{{\text{s}}} }}{L} - \frac{28}{9}(T_{{\text{s}}} - T_{{\text{r}}} )\sqrt {\frac{{\lambda_{{{\text{layer}}}} \rho_{{{\text{layer}}}} c_{{{\text{layer}}}} }}{{\uppi }}} \overline{V}_{1} [\frac{{(t - t_{{\text{C}}} )^{\frac{3}{2}} - (t_{{{\text{CR4}}}} - t_{{\text{C}}} )^{\frac{3}{2}} }}{{t - t_{{{\text{CR4}}}} }} - \sqrt {t - t_{{{\text{CR4}}}} } ] \\ & \quad = \rho c(T_{s} - T_{r} )h\overline{V} + \frac{28}{9}(T_{{\text{s}}} - T_{{\text{r}}} )\sqrt {\frac{{\lambda_{{{\text{layer}}}} \rho_{{{\text{layer}}}} c_{{{\text{layer}}}} }}{{\uppi }}} \overline{V} \sqrt {t - t_{{{\text{CR4}}}} } ,{ (}t_{{{\text{CR4}}}} < t < t_{{{\text{ER}}}} ) \\ \end{aligned}$$

By solving Eq. (34) (see Appendix B), we obtain the expression of the front distance of steam chamber and oil production rate:

35 $$x = \frac{{A_{3} - B_{3} [\frac{{(t - t_{{\text{C}}} )^{\frac{3}{2}} - (t_{{{\text{CR4}}}} - t_{{\text{C}}} )^{\frac{3}{2}} }}{{t - t_{{{\text{CR4}}}} }} - \sqrt {t - t_{{{\text{CR4}}}} } ]}}{{C + D\sqrt {t - t_{{{\text{CR4}}}} } }}(t - t_{{{\text{CR4}}}} ),{ (}t_{{{\text{CR4}}}} < t < t_{{{\text{ER}}}} )$$

where, $$A_{3} = \frac{{\chi \overline{q}_{{\text{s}}} H_{{\text{s}}} }}{L}$$,$$B_{3} = \frac{28}{9}(T_{{\text{s}}} - T_{{\text{r}}} )\sqrt {\frac{{\lambda_{{{\text{layer}}}} \rho_{{{\text{layer}}}} c_{{{\text{layer}}}} }}{{\uppi }}} \overline{V}_{1}$$,$$C = \rho c(T_{{\text{s}}} - T_{{\text{r}}} )h$$,

$$D = \frac{28}{9}(T_{{\text{s}}} - T_{{\text{r}}} )\sqrt {\frac{{\lambda_{{{\text{layer}}}} \rho_{{{\text{layer}}}} c_{{{\text{layer}}}} }}{{\uppi }}}$$.

36 $$q = \frac{{A_{3}{\prime} - B_{3}{\prime} (\sqrt {t - t_{{\text{C}}} } - \sqrt {t_{{{\text{CR4}}}} - t_{{\text{C}}} )} - D_{1} \overline{V} \sqrt {t - t_{{{\text{CR4}}}} } }}{{C_{1} }},{ (}t_{{{\text{CR4}}}} < t < t_{{{\text{ER}}}} )$$

where, $$A_{3}{\prime} = \frac{{\chi q_{{\text{s,i}}} H_{{\text{s}}} }}{L}$$, $$B_{3}{\prime} = \frac{14}{3}(T_{{\text{s}}} - T_{{\text{r}}} )\sqrt {\frac{{\lambda_{{{\text{layer}}}} \rho_{{{\text{layer}}}} c_{{{\text{layer}}}} }}{{\uppi }}} \overline{V}_{1}$$,$$C_{1} = \frac{{\rho c(T_{{\text{s}}} - T_{{\text{r}}} {)}}}{{\phi \Delta S_{{\text{o}}} \rho_{{\text{o}}} }}$$, $$D_{1} = \frac{14}{3}(T_{{\text{s}}} - T_{{\text{r}}} )\sqrt {\frac{{\lambda_{{{\text{layer}}}} \rho_{{{\text{layer}}}} c_{{{\text{layer}}}} }}{{\uppi }}}$$.

### Mathematic model of the second rising stage

During the second rising stage, we also consider that heat loss is consumed at both the interlayer and the side interface of steam chamber, so the heat loss can be divided into two parts.

According to Eq. (19), the interlayer heat loss during the second rising stage of sub-chamber can be expressed as

37 $$q_{{{\text{layer}}}} = 4(T_{{\text{s}}} - T_{{\text{r}}} )\sqrt {\frac{{\lambda_{{{\text{layer}}}} \rho_{{{\text{layer}}}} c_{{{\text{layer}}}} }}{{\uppi }}} \overline{V}_{2} (\sqrt {t - t_{{\text{C}}} } - \sqrt {t - t_{{{\text{ER}}}} } ),{ (}t_{{{\text{ER}}}} < t < t_{{\text{D}}} )$$

38 $$\overline{V}_{2} = \frac{{w_{{\text{c}}} }}{{2(t_{{{\text{ER}}}} - t_{{\text{C}}} )}}$$

where, $\overline{V}_{2}$ is average movement speed of sub-chamber from *t*_C_ to *t*_ER_, m/d; and *w*_c_ is interlayer width, m.

The rate of heat consumed per unit horizontal well length of sub-chamber expansion is calculated by

39 $$q_{{{\text{in}}}} = 4{\uppi }\rho c(T_{{\text{s}}} - T_{{\text{r}}} )\eta a_{2} \frac{{{\text{d}}a_{{2}} }}{{{\text{d}}t}},{ (}t_{{{\text{ER}}}} < t < t_{{\text{D}}} )$$

where, *a*_2_ is major radius of oval chamber in the second rising stage, m.

Combining Eqs. (3), (14), (18), (21) and (37)–(39), we obtain

40 $$\begin{aligned} & \frac{{\chi q_{{\text{s}}} H_{{\text{s}}} }}{L} - 4{\uppi }\rho c(T_{{\text{s}}} - T_{{\text{r}}} )\eta a_{2} \frac{{{\text{d}}a_{2} }}{{{\text{d}}t}} \\ & \quad = \frac{14}{3}(T_{{\text{s}}} - T_{{\text{r}}} )\sqrt {\frac{{\lambda_{{{\text{layer}}}} \rho_{{{\text{layer}}}} c_{{{\text{layer}}}} }}{{\uppi }}} \overline{V}_{2} (\sqrt {t - t_{{\text{C}}} } - \sqrt {t - t_{{{\text{ER}}}} } ),{ (}t_{{{\text{ER}}}} < t < t_{{\text{D}}} ) \\ \end{aligned}$$

Let $A_{4} = \frac{{\chi q_{{\text{s}}} H_{{\text{s}}} }}{L}$, $B_{4} = 4{\uppi }\rho c\left( {T_{{\text{s}}} - T_{{\text{r}}} } \right)$, $C = \frac{14}{3}(T_{{\text{s}}} - T_{{\text{r}}} )\sqrt {\frac{{\lambda_{{{\text{layer}}}} \rho_{{{\text{layer}}}} c_{{{\text{layer}}}} }}{{\uppi }}} \overline{V}_{2}$, and substitute these equations into Eq. (40):

41 $$A_{4} - C(\sqrt {t - t_{{\text{C}}} } - \sqrt {t - t_{{{\text{ER}}}} } {) = }B_{4} \beta a_{{\text{s}}} \frac{{{\text{d}}a_{{\text{s}}} }}{{{\text{d}}t}},{ (}t_{{{\text{ER}}}} < t < t_{{\text{D}}} )$$

By integrating Eq. (41), the major radius of sub-chamber is calculated by

42 $$a_{2} = \sqrt {2\int_{{t_{{{\text{ER}}}} }}^{t} {\frac{{A_{4} - C(\sqrt {t - t_{{\text{C}}} } - \sqrt {t - t_{{{\text{ER}}}} } )}}{{\eta B_{4} }}{\text{d}}t} } ,{ (}t_{{{\text{ER}}}} < t < t_{{\text{D}}} )$$

Then, the perpendicular front distance can be obtained by

43 $$h_{2} = 2a_{2} = 2\sqrt {2\int_{{t_{{{\text{ER}}}} }}^{t} {\frac{{A_{4} - C(\sqrt {t - t_{{\text{C}}} } - \sqrt {t - t_{{{\text{ER}}}} } )}}{{\eta B_{4} }}{\text{d}}t} } ,{ (}t_{{{\text{ER}}}} < t < t_{{\text{D}}} )$$

where, *h*_2_ is rising height of oval sub-chamber, m.

Based on the material balance principle, the oil production rate per unit of horizontal well length before the sub-chamber reaches the cap rock can be calculated by

44 $$q = 4{\uppi }\rho_{{\text{o}}} \phi \Delta S_{{\text{o}}} \eta a_{{\text{s}}} \frac{{{\text{d}}a_{{\text{s}}} }}{{{\text{d}}t}},{ (}t_{{{\text{ER}}}} < t < t_{{\text{D}}} )$$

Substituting Eq. (42) into Eq. (44), we obtain

45 $$q = \frac{{4{\uppi }\rho_{{\text{o}}} \phi \Delta S_{{\text{o}}} (A_{4} - C(\sqrt {t - t_{{\text{C}}} } - \sqrt {t - t_{{{\text{ER}}}} } )}}{{B_{4} }},{ (}t_{{{\text{ER}}}} < t < t_{{\text{D}}} )$$

where, $$A_{4} = \frac{{\chi q_{{\text{s}}} H_{{\text{s}}} }}{L}$$,$$B_{4} = 4{\uppi }\rho c\left( {T_{{\text{s}}} - T_{{\text{r}}} } \right)$$,$$C = \frac{14}{3}(T_{{\text{s}}} - T_{{\text{r}}} )\sqrt {\frac{{\lambda_{{{\text{layer}}}} \rho_{{{\text{layer}}}} c_{{{\text{layer}}}} }}{{\uppi }}} \overline{V}_{2}$$.

### Mathematic model of the second lateral expansion stage

- The development process of area 1 of the sub-chamber.
- The early lateral expansion period of area 1.

The consumed heat per unit horizontal well length of the early lateral expansion period of area 1 is calculated by

46 $$Q_{{{\text{in}}}} = \rho c(T_{{\text{s}}} - T_{{\text{r}}} )(H - h){\text{d}}x$$

Take the derivative of Eq. (46) with respect to *t*:

47 $$q_{{{\text{in}}}} = \frac{{{\text{d}}Q_{{{\text{in}}}} }}{{{\text{d}}t}} = \rho c(T_{{\text{s}}} - T_{{\text{r}}} )(H - h)\frac{{{\text{d}}x}}{{{\text{d}}t}},{ (}t_{{\text{D}}} < t < t_{DL3} )$$

where, *H* is perpendicular distance from the production well to the cap rock, m.

After the sub-chamber bypasses the interlayer, the heat loss of the interlayer gradually decreases. After the sub-chamber reaches to the cap rock, the heat loss of the interlayer is far smaller than that of the cap rock, so we ignore the heat loss of the interlayer in our model and only consider the heat losses of the cap rock and the side interface of sub-chamber. The total heat loss in this period can be expressed by

48 $$q_{{{\text{loss}}}} = q_{{{\text{cap}}}} + q_{{{\text{side}}}}$$

According to Eq. (19), the heat loss of the cap rock is written by

49 $$q_{{{\text{cap}}}} = 4\int_{{t_{{\text{D}}} }}^{t} {(T_{{\text{s}}} - T_{{\text{r}}} )\sqrt {\frac{{\lambda_{{{\text{cap}}}} \rho_{{{\text{cap}}}} c_{{{\text{cap}}}} }}{{{\uppi }(t - \tau )}}} } \frac{{{\text{d}}x}}{{{\text{d}}\tau }}{\text{d}}\tau ,{ (}t_{{\text{D}}} < t < t_{DL3} )$$

where, *t*_D_ is time of steam chamber reaching to point D, d.

Combining Eqs. (3), (14), (21) and (46)–(49), we obtain

50 $$\frac{{\chi q_{{\text{s}}} H_{{\text{s}}} }}{L} = \frac{14}{3}\int_{{t_{{\text{D}}} }}^{t} {(T_{{\text{s}}} - T_{{\text{r}}} )\sqrt {\frac{{\lambda_{{{\text{cap}}}} \rho_{{{\text{cap}}}} c_{{{\text{cap}}}} }}{{{\uppi }(t - \tau )}}} } \frac{{{\text{d}}x}}{{{\text{d}}\tau }}{\text{d}}\tau + \rho c(T_{{\text{s}}} - T_{{\text{r}}} )(H - h)\frac{{{\text{d}}x}}{{{\text{d}}t}}{, (}t_{{\text{D}}} < t < t_{{{\text{DL3}}}} )$$

By solving Eq. (50) (see Appendix C), we obtain the expression of the front distance of sub-chamber:

51 $$x = \int_{{t_{{\text{D}}} }}^{t} {\frac{{A_{5} }}{C}{\text{e}}^{{[\frac{{B_{5} }}{C}\Gamma (0.5)]^{2} \tau }} {\text{erfc}}[\frac{{B_{5} }}{C}\Gamma (0.5)\sqrt \tau ]{\text{d}}\tau } ,{ (}t_{{\text{D}}} < t < t_{{{\text{DL3}}}} )$$

Based on the material balance principle, the oil production rate per unit of horizontal well length can be calculated by

52 $$q = \rho_{{\text{o}}} \phi \Delta S_{{\text{o}}} {(}H{ - }h{)}\frac{dx}{{dt}},{ (}t_{{\text{D}}} < t < t_{{{\text{DL3}}}} )$$

Substituting Eq. (51) into Eq. (52), we obtain

53 $$q = \rho_{{\text{o}}} \phi \Delta S_{{\text{o}}} (H - h)\frac{{A_{5} }}{C}{\text{e}}^{{[\frac{{B_{5} }}{C}\Gamma (0.5)]^{2} (t - t_{{\text{D}}} )}} {\text{erfc}}[\frac{{B_{5} }}{C}\Gamma (0.5)\sqrt {t - t_{{\text{D}}} } ],{ (}t_{{\text{D}}} < t < t_{{{\text{DL3}}}} )$$

where, $$A_{5} = \frac{{\chi q_{{\text{s}}} H_{{\text{s}}} }}{L}$$,$$B_{5} = \frac{14}{3}(T_{{\text{s}}} - T_{{\text{r}}} )\sqrt {\frac{{\lambda_{{{\text{cap}}}} \rho_{{{\text{cap}}}} c_{{{\text{cap}}}} }}{{\uppi }}}$$,$$C = \rho c(T_{{\text{s}}} - T_{{\text{r}}} )(H - h)$$.

(b) The middle lateral expansion period of area 1.

After the steam chamber moves to point D_L3_ (Fig. 8), the latent heat released by steam per unit horizontal well length during this period can be calculated by

54 $$Q_{{{\text{inject}}}} = \frac{{\chi H_{{\text{s}}} }}{2L}\overline{q}_{{\text{s}}} (t - t_{{{\text{DL3}}}} ),{ (}t_{{{\text{DL3}}}} < t < t_{{\text{M}}} )$$

55 $$\overline{q}_{{\text{s}}} = \frac{{\sum\limits_{{i = t_{{{\text{DL3}}}} }}^{t} {q_{{\text{s,i}}} } }}{{t - t_{{{\text{DL3}}}} }},{ (}t_{{{\text{DL3}}}} < t < t_{{\text{M}}} )$$

where, *t*_DL3_ is time of steam chamber reaching to point D_L3_, day; and *t*_M_ is time of steam chamber reaching to point M, day.

The heat consumed per unit horizontal well length of sub-chamber expansion can be calculated by

56 $$Q_{{{\text{in}}}} = \rho c(T_{{\text{s}}} - T_{{\text{r}}} )(H - h)\overline{V}_{{\text{a}}} (t - t_{{{\text{DL3}}}} ),{ (}t_{{{\text{DL3}}}} < t < t_{{\text{M}}} )$$

where, $\overline{V}_{{\text{a}}}$ is the average movement speed of sub-chamber from point D_L3_ to point D_M_.

According to Eq. (19), the heat loss of the cap rock is expressed as

57 $$q_{{{\text{cap}}}} = 4(T_{{\text{s}}} - T_{{\text{r}}} )\sqrt {\frac{{\lambda_{{{\text{cap}}}} \rho_{{{\text{cap}}}} c_{{{\text{cap}}}} }}{{\uppi }}} [\overline{V}_{{3}} (\sqrt {t - t_{{\text{D}}} } - \sqrt {t - t_{{{\text{DL3}}}} } ) + \overline{V}_{a} \sqrt {t - t_{{{\text{DL3}}}} } ],{ (}t_{{{\text{DL3}}}} < t < t_{{\text{M}}} )$$

58 $$\overline{V}_{3} = \frac{{x_{{{\text{DDL3}}}} }}{{t_{{{\text{DL3}}}} - t_{{\text{D}}} }}$$

59 $$x_{{{\text{DDL3}}}} = \beta (H - h)$$

where, $\overline{V}_{{3}}$ is average movement speed of sub-chamber from point D to point D_L3_, m/day; and *x*_DDL3_ is length of line DD_L3_, m.

The heat loss per unit horizontal well length of steam chamber expansion can be calculated by

60 $$Q_{{{\text{loss}}}} = \int_{{t_{{{\text{DL3}}}} }}^{t} {q_{{{\text{loss}}}} {\text{d}}t} ,{ (}t_{{{\text{DL3}}}} < t < t_{{\text{M}}} )$$

Combining Eqs. (21), (29), (48) and (54)–(60), we obtain

61 $$\begin{gathered} \frac{{\chi \overline{q}_{{\text{s}}} H_{{\text{s}}} }}{2L} - \frac{28}{9}(T_{{\text{s}}} - T_{{\text{r}}} )\overline{V}_{3} \sqrt {\frac{{\lambda_{{{\text{cap}}}} \rho_{{{\text{cap}}}} c_{{{\text{cap}}}} }}{{\uppi }}} [\frac{{(t - t_{{\text{D}}} )^{\frac{3}{2}} - (t_{{{\text{DL3}}}} - t_{{\text{D}}} )^{\frac{3}{2}} }}{{t - t_{{{\text{DL3}}}} }} - \sqrt {t - t_{{{\text{DL3}}}} } ] \hfill \\ = \rho c(T_{{\text{s}}} - T_{{\text{r}}} )(H - h)\overline{V}_{{\text{a}}} + \frac{28}{9}(T_{{\text{s}}} - T_{{\text{r}}} )\sqrt {\frac{{\lambda_{{{\text{cap}}}} \rho_{{{\text{cap}}}} c_{{{\text{cap}}}} }}{{\uppi }}} \overline{V}_{{\text{a}}} \sqrt {t - t_{{{\text{DL3}}}} } ,{ (}t_{{{\text{DL3}}}} < t < t_{{\text{M}}} ) \hfill \\ \end{gathered}$$

By solving Eq. (61) (see Appendix D), we obtain the expression of the front distance of sub-chamber and oil production rate:

62 $$x = \frac{{A_{6} - B_{6} [\frac{{(t - t_{{\text{D}}} )^{\frac{3}{2}} - (t_{{{\text{DL3}}}} - t_{{\text{D}}} )^{\frac{3}{2}} }}{t} - \sqrt {t - t_{{{\text{DL3}}}} } ]}}{{C + D\sqrt {t - t_{{{\text{DL3}}}} } }}(t - t_{{{\text{DL3}}}} ),{ (}t_{{{\text{DL3}}}} < t < t_{{\text{M}}} )$$

where, $$A_{6} = \frac{{\chi \overline{q}_{{\text{s}}} H_{{\text{s}}} }}{2L}$$, $$B_{6} = \frac{28}{9}(T_{{\text{s}}} - T_{{\text{r}}} )\sqrt {\frac{{\lambda_{{{\text{cap}}}} \rho_{{{\text{cap}}}} c_{{{\text{cap}}}} }}{{\uppi }}} \overline{V}_{3}$$, $$C = \rho c(T_{{\text{s}}} - T_{{\text{r}}} )(H - h)$$, $$D = \frac{28}{9}(T_{{\text{s}}} - T_{{\text{r}}} )\sqrt {\frac{{\lambda_{{{\text{cap}}}} \rho_{{{\text{cap}}}} c_{{{\text{cap}}}} }}{{\uppi }}}$$.

63 $$q_{a} = \frac{{A_{6}{\prime} - B_{6}{\prime} (\sqrt {t - t_{{\text{D}}} } - \sqrt {t - t_{{{\text{DL3}}}} } ) - D_{1} \overline{V}_{a} \sqrt {t - t_{{{\text{DL3}}}} } }}{{C_{1} }},{ (}t_{{{\text{DL3}}}} < t < t_{{\text{M}}} )$$

where, $$A_{6}{\prime} = \frac{{\chi q_{{\text{s,i}}} H_{{\text{s}}} }}{L}$$, $$B_{6}{\prime} = \frac{14}{3}(T_{{\text{s}}} - T_{{\text{r}}} )\sqrt {\frac{{\lambda_{{{\text{layer}}}} \rho_{{{\text{layer}}}} c_{{{\text{layer}}}} }}{{\uppi }}} \overline{V}_{3}$$, $$C_{1} = \frac{{\rho c(T_{{\text{s}}} - T_{{\text{r}}} {)}}}{{\phi \Delta S_{{\text{o}}} \rho_{{\text{o}}} }}$$, $$D_{1} = \frac{14}{3}(T_{{\text{s}}} - T_{{\text{r}}} )\sqrt {\frac{{\lambda_{{{\text{layer}}}} \rho_{{{\text{layer}}}} c_{{{\text{layer}}}} }}{{\uppi }}}$$.

(c) The late downward expansion period of area 1.

According to Butler's derivation^2^, the oil production rate per unit of horizontal well when the sub-chamber expands downward is calculated by

64 $$q_{{\text{a}}} = 2\left[ {\sqrt{\frac{3}{2}} - \left( {\frac{{t - t_{{\text{M}}} }}{{w_{{\text{C}}} }}} \right)^{2} \frac{kg\alpha }{{\phi {\Delta }s_{{\text{o}}} mv_{{\text{s}}} (H - h)}}\sqrt{\frac{2}{3}} } \right]\sqrt {\frac{{kg\alpha \phi {\Delta }S_{{\text{o}}} (H - h)}}{{mv_{{\text{s}}} }}} ,{ (}t_{{\text{M}}} { < }t{ < }t_{{{\text{ML}}}} )$$

where, *k* is effective permeability of oil flow, m^2^; *α* is thermal diffusivity of reservoir rock, m^2^/day; *m* is a coefficient constant; *v*_so_ is kinematic viscosity of oil at the temperature inside steam chamber, m^2^/day; *q*_a_ is oil production rate per unit of horizontal well in area 1, m^3^/(m day).

The released heat rate per unit of horizontal well length in area 1 can be calculated by

65 $$q_{{{\text{inject}}}} = \frac{{\chi q_{{\text{s,a}}} H_{{\text{s}}} }}{L}$$

where, *q*_s,a_ is steam flow rate in area 1, kg/day.

The consumed heat rate per unit horizontal well length of sub-chamber expansion in area 1 is calculated by

66 $$q_{{{\text{in}}}} = \rho c(T_{{\text{s}}} - T_{{\text{r}}} )\frac{{q_{{\text{a}}} }}{{\phi \Delta S_{{\text{o}}} }}$$

According to Eq. (19), the heat loss of the cap rock is expressed as

67 $$q_{{{\text{cap}}}} = 2\int_{{t_{{\text{D}}} }}^{{t_{{\text{M}}} }} {(T_{{\text{s}}} - T_{{\text{r}}} )\sqrt {\frac{{\lambda_{{{\text{cap}}}} \rho_{{{\text{cap}}}} c_{{{\text{cap}}}} }}{{{\uppi }(t - \tau )}}} } \overline{V}_{{4}} d\tau ,{ (}t_{{\text{M}}} { < }t{ < }t_{{{\text{ML}}}} )$$

68 $$\overline{V}_{4} = \frac{{w_{{\text{c}}} }}{{2(t_{{\text{M}}} - t_{{\text{D}}} )}}$$

where, $\overline{V}_{{4}}$ is average speed of sub-chamber from point D to point M, m/day.

Combining Eqs. (14), (48) and (65)–(68), we obtain

69 $$\frac{{\chi q_{{\text{s,a}}} H_{{\text{s}}} }}{L} = \rho c(T_{{\text{s}}} - T_{{\text{r}}} )\frac{{q_{{\text{a}}} }}{{\phi \Delta S_{{\text{o}}} }} + \frac{7}{3}\int_{{t_{{\text{D}}} }}^{{t_{{\text{M}}} }} {(T_{{\text{s}}} - T_{{\text{r}}} )\sqrt {\frac{{\lambda_{{{\text{cap}}}} \rho_{{{\text{cap}}}} c_{{{\text{cap}}}} }}{{{\uppi }(t - \tau )}}} } \overline{V}_{{4}} {\text{d}}\tau ,{ (}t_{{\text{M}}} { < }t{ < }t_{{{\text{ML}}}} )$$

Change the form of Eq. (69), we can get

70 $$q_{{\text{s,a}}} = L[\rho c(T_{{\text{s}}} - T_{{\text{r}}} )\frac{{q_{{\text{a}}} }}{{\phi \Delta S_{{\text{o}}} }} + \frac{7}{3}\int_{{t_{{\text{D}}} }}^{{t_{M} }} {(T_{{\text{s}}} - T_{{\text{r}}} )\sqrt {\frac{{\lambda_{{{\text{cap}}}} \rho_{{{\text{cap}}}} c_{{{\text{cap}}}} }}{{{\uppi }(t - \tau )}}} \overline{V}_{{4}} {\text{d}}\tau ]/(\chi H_{{\text{s}}} )} {, (}t_{{\text{M}}} { < }t{ < }t_{{{\text{ML}}}} )$$

Based on the material balance principle, the oil production rate per unit of horizontal well length can be calculated by

71 $$q_{{\text{a}}} = \phi \Delta S_{{\text{o}}} \frac{{w_{{\text{C}}} }}{2}\frac{{{\text{d}}y}}{{{\text{d}}t}}{, (}t_{{\text{M}}} { < }t{ < }t_{{{\text{ML}}}} )$$

By integrating Eq. (71), the perpendicular front distance can be obtained by

72 $$y = \int_{{t_{{\text{M}}} }}^{t} {\frac{{2q_{{\text{a}}} }}{{\phi \Delta S_{{\text{o}}} w_{{\text{c}}} }}} dt,{ (}t_{{\text{M}}} { < }t{ < }t_{{{\text{ML}}}} )$$

where, *y* is perpendicular front distance, m.

(i) The development process of area 2 of the sub-chamber.

- The early lateral expansion period of area 2.

Before the sub-chamber moves to point D_M_, the development process of area 2 is the same as that of area 1 (see Figs. 7 and 8); therefore, the mathematical model for area 2 is the same as that for area 1, and it is omitted here.

(b) The middle lateral expansion period of area 2.

After the sub-chamber moves to point D_M_ (Fig. 10), the latent heat released by steam per unit horizontal well length during this period can be calculated by

73 $$Q_{{{\text{inject}}}} = \frac{{\chi \overline{q}_{{\text{s,b}}} H_{{\text{s}}} }}{L}(t - t_{{{\text{DM}}}} ),{ (}t_{{{\text{DM}}}} { < }t{ < }t_{{{\text{DR5}}}} )$$

74 $$\overline{q}_{{\text{s,b}}} = \frac{{\sum\limits_{{i = t_{{{\text{DM}}}} }}^{t} {q_{{{\text{s,b,}}i}} } }}{{t - t_{{{\text{DM}}}} }},{ (}t_{{{\text{DM}}}} { < }t{ < }t_{{{\text{DR5}}}} )$$

where, $\overline{q}_{{\text{s,b}}}$ is average steam flow rate in area 2, kg/day; *q*_s,b,*i*_ is steam flow rate on the *i*th day in area 2, kg/day.

The heat consumed per unit horizontal well length of sub-chamber expansion can be calculated by

75 $$Q_{{{\text{in}}}} = \rho c(T_{{\text{s}}} - T_{{\text{r}}} )(H - h)\overline{V}_{{\text{b}}} (t - t_{{{\text{DM}}}} ),{ (}t_{{{\text{DM}}}} { < }t{ < }t_{{{\text{DR5}}}} )$$

where, $\overline{V}_{{\text{b}}}$ is average movement speed of sub-chamber from point D_M_ to point D_R5_, m/day.

According to Eq. (19), the heat loss of the cap rock is expressed as

76 $$q_{{{\text{cap}}}} = 4(T_{{\text{s}}} - T_{{\text{r}}} {)}\sqrt {\frac{{\lambda_{{{\text{cap}}}} \rho_{{{\text{cap}}}} c_{{{\text{cap}}}} }}{{\uppi }}} [\overline{V}_{{_{3} }} (\sqrt {t - t_{{\text{D}}} } - \sqrt {t - t_{{{\text{DM}}}} } ) + \overline{V}_{{_{{\text{b}}} }} \sqrt {t - t_{{{\text{DM}}}} } ],{ (}t_{{{\text{DM}}}} { < }t{ < }t_{{{\text{DR5}}}} )$$

The heat loss per unit horizontal well length of steam chamber expansion can be calculated by

77 $$Q_{{{\text{loss}}}} = \int_{{t_{{{\text{DM}}}} }}^{t} {q_{{{\text{loss}}}} {\text{d}}t} ,{ (}t_{{{\text{DM}}}} < t < t_{{{\text{DR5}}}} )$$

Combining Eqs. (21), (29), (48) and (73)−(77), we obtain

78 $$\begin{aligned} & \frac{{\chi \overline{q}_{{\text{s,b}}} H_{{\text{s}}} }}{L} - \frac{28}{9}(T_{{\text{s}}} - T_{{\text{r}}} )\overline{V}_{3} \sqrt {\frac{{\lambda_{{{\text{cap}}}} \rho_{{{\text{cap}}}} c_{{{\text{cap}}}} }}{{\uppi }}} [\frac{{(t - t_{{\text{D}}} )^{\frac{3}{2}} - (t_{{{\text{DM}}}} - t_{{\text{D}}} )^{\frac{3}{2}} }}{{t - t_{{{\text{DM}}}} }} - \sqrt {t - t_{{{\text{DM}}}} } ] \\ & \quad = \rho c(T_{{\text{s}}} - T_{{\text{r}}} )(H - h)\overline{V}_{{\text{b}}} + \frac{28}{9}(T_{{\text{s}}} - T_{{\text{r}}} )\sqrt {\frac{{\lambda_{{{\text{cap}}}} \rho_{{{\text{cap}}}} c_{{{\text{cap}}}} }}{{\uppi }}} \overline{V}_{{\text{b}}} \sqrt {t - t_{{{\text{DM}}}} } ,{ (}t_{{{\text{DM}}}} { < }t{ < }t_{{{\text{DR5}}}} ) \\ \end{aligned}$$

By solving Eq. (78) (see Appendix E), we obtain the expression of the front distance of sub-chamber and oil production rate:

79 $$x = \frac{{A_{7} - B_{7} [\frac{{(t - t_{{\text{D}}} )^{\frac{3}{2}} - (t_{{{\text{DM}}}} - t_{{\text{D}}} )^{\frac{3}{2}} }}{{t - t_{{{\text{DM}}}} }} - \sqrt {t - t_{{{\text{DM}}}} } ]}}{{C + D\sqrt {t - t_{{{\text{DM}}}} } }}(t - t_{{{\text{DM}}}} ),{ (}t_{{{\text{DM}}}} { < }t{ < }t_{{{\text{DR5}}}} )$$

where, $$A_{7} = \frac{{\chi \overline{q}_{{\text{s,b}}} H_{{\text{s}}} }}{L}$$, $$B_{7} = \frac{28}{9}(T_{{\text{s}}} - T_{{\text{r}}} )\sqrt {\frac{{\lambda_{{{\text{cap}}}} \rho_{{{\text{cap}}}} c_{{{\text{cap}}}} }}{{\uppi }}} \overline{V}_{3}$$, $$C = \rho c(T_{{\text{s}}} - T_{{\text{r}}} )(H - h)$$, $$D = \frac{28}{9}(T_{{\text{s}}} - T_{{\text{r}}} )\sqrt {\frac{{\lambda_{{{\text{cap}}}} \rho_{{{\text{cap}}}} c_{{{\text{cap}}}} }}{{\uppi }}}$$.

80 $$q_{{\text{b}}} = \frac{{A_{7}{\prime} - B_{7}{\prime} (\sqrt {t - t_{{\text{D}}} } - \sqrt {t - t_{{{\text{DM}}}} } ) - D_{1} \overline{V}_{{\text{b}}} \sqrt {t - t_{{{\text{DM}}}} } }}{{C_{1} }},{ (}t_{{{\text{DM}}}} { < }t{ < }t_{{{\text{DR5}}}} )$$

where, *q*_b_ is oil production rate per horizontal well length in area 2, kg/(m·d);$A_{7}{\prime} = \frac{{\chi q_{{\text{s,b,i}}} H_{{\text{s}}} }}{L}$, $B_{7}{\prime} = \frac{14}{3}(T_{{\text{s}}} - T_{{\text{r}}} )\sqrt {\frac{{\lambda_{{{\text{cap}}}} \rho_{{{\text{cap}}}} c_{{{\text{cap}}}} }}{{\uppi }}} \overline{V}_{{_{3} }}$, $C_{1} = \frac{{\rho c(T_{{\text{s}}} - T_{{\text{r}}} )}}{{\rho_{{\text{o}}} \phi \Delta S_{{\text{o}}} }}$, $D_{1} = \frac{14}{3}(T_{{\text{s}}} - T_{{\text{r}}} )\sqrt {\frac{{\lambda_{{{\text{cap}}}} \rho_{{{\text{cap}}}} c_{{{\text{cap}}}} }}{{\uppi }}}$.

(c) The late lateral expansion period of area 2.

After the sub-chamber moves to point D_R5_ (Fig. 9), the latent heat released by steam per unit horizontal well length during this period can be calculated by

81 $$Q_{{{\text{inject}}}} = \frac{{\chi \overline{q}_{{\text{s,b}}} H_{{\text{s}}} }}{L}(t - t_{{{\text{DR5}}}} ),{ (}t_{{{\text{DR5}}}} { < }t{ < }t_{{{\text{FR}}}} )$$

82 $$\overline{q}_{{\text{s,b}}} = \frac{{\sum\limits_{{i = t_{{{\text{DR5}}}} }}^{t} {q_{{{\text{s,b,}}i}} } }}{{t - t_{{{\text{DR5}}}} }},{ (}t_{{{\text{DR5}}}} { < }t{ < }t_{{{\text{FR}}}} )$$

The heat consumed per unit horizontal well length of sub-chamber expansion can be calculated by

83 $$Q_{{{\text{in}}}} = \rho c(T_{{\text{s}}} - T_{{\text{r}}} )H\overline{V}_{b2} (t - t_{{{\text{DR5}}}} ),{ (}t_{{{\text{DR5}}}} < t < t_{{{\text{FR}}}} )$$

where, $\overline{V}_{{{\text{b2}}}}$ is average movement speed of sub-chamber from point D_R5_ to point F_R_, m/d; and *t*_FR_ is time of steam chamber reaching to point F_R_, d.

According to Eq. (19), the heat loss of the cap rock is expressed as

84 $$q_{{{\text{cap}}}} = 4(T_{{\text{s}}} - T_{{\text{r}}} )\sqrt {\frac{{\lambda_{{{\text{cap}}}} \rho_{{{\text{cap}}}} c_{{{\text{cap}}}} }}{{\uppi }}} [\overline{V}_{5} (\sqrt {t - t_{{\text{D}}} } - \sqrt {t - t_{{{\text{DR5}}}} } ) + \overline{V}_{{{\text{b2}}}} \sqrt {t - t_{{{\text{DR5}}}} } ],{ (}t_{{{\text{DR5}}}} { < }t{ < }t_{{{\text{FR}}}} )$$

85 $$\overline{V}_{5} = \frac{{x_{{{\text{DDR5}}}} }}{{t_{{{\text{DR5}}}} - t_{{\text{D}}} }}$$

where, $\overline{V}_{5}$ is average speed of sub-chamber from point D to point D_R5_, m/day; *x*_DDR5_ is length of line DD_R5_, m.

The heat loss per unit horizontal well length of steam chamber expansion can be calculated by

86 $$Q_{{{\text{loss}}}} = \int_{{t_{{{\text{DR5}}}} }}^{t} {q_{{{\text{loss}}}} {\text{d}}t} ,{ (}t_{{{\text{DR5}}}} < t < t_{{{\text{FR}}}} )$$

Combining Eqs. (21), (29), (48) and (81)−(86), we obtain

87 $$\begin{aligned} & \frac{{\chi \overline{q}_{{\text{s,b}}} H_{{\text{s}}} }}{L} - \frac{28}{9}(T_{{\text{s}}} - T_{{\text{r}}} )\overline{V}_{5} \sqrt {\frac{{\lambda_{{{\text{cap}}}} \rho_{{{\text{cap}}}} c_{{{\text{cap}}}} }}{{\uppi }}} [\frac{{(t - t_{{\text{D}}} )^{\frac{3}{2}} - (t_{{{\text{DR5}}}} - t_{{\text{D}}} )^{\frac{3}{2}} }}{{t - t_{{{\text{DR5}}}} }} - \sqrt {t - t_{{{\text{DR5}}}} } ] \\ & \quad = \rho c(T_{{\text{s}}} - T_{{\text{r}}} )H\overline{V}_{{{\text{b2}}}} + \frac{28}{9}(T_{{\text{s}}} - T_{{\text{r}}} )\sqrt {\frac{{\lambda_{{{\text{cap}}}} \rho_{{{\text{cap}}}} c_{{{\text{cap}}}} }}{{\uppi }}} \overline{V}_{{{\text{b2}}}} \sqrt {t - t_{{{\text{DR5}}}} } ,{ (}t_{{{\text{DR5}}}} { < }t{ < }t_{{{\text{FR}}}} ) \\ \end{aligned}$$

By solving Eq. (87) (see Appendix F), we obtain the expression of the front distance of steam chamber and oil production rate:

88 $$x = \frac{{A_{8} - B_{8} [\frac{{(t - t_{{\text{D}}} )^{\frac{3}{2}} - (t_{{{\text{DR5}}}} - t_{{\text{D}}} )^{\frac{3}{2}} }}{{t - t_{{{\text{DR5}}}} }} - \sqrt {t - t_{{{\text{DR5}}}} } ]}}{{C + D\sqrt {t - t_{{{\text{DR5}}}} } }}(t - t_{{{\text{DR5}}}} ),{ (}t_{{{\text{DR5}}}} { < }t{ < }t_{{{\text{FR}}}} )$$

where, $$A_{8} = \frac{{\chi \overline{q}_{{\text{s,b}}} H_{{\text{s}}} }}{L}$$, $$B_{8} = \frac{28}{9}(T_{{\text{s}}} - T_{{\text{r}}} )\sqrt {\frac{{\lambda_{{{\text{cap}}}} \rho_{{{\text{cap}}}} c_{{{\text{cap}}}} }}{{\uppi }}} \overline{V}_{5}$$, $$C = \rho c(T_{{\text{s}}} - T_{{\text{r}}} )H$$, $$D = \frac{28}{9}(T_{{\text{s}}} - T_{{\text{r}}} )\sqrt {\frac{{\lambda_{{{\text{cap}}}} \rho_{{{\text{cap}}}} c_{{{\text{cap}}}} }}{{\uppi }}}$$.

89 $$q_{{\text{b}}} = \frac{{A_{8}{\prime} - B_{8}{\prime} (\sqrt {t - t_{{\text{D}}} } - \sqrt {t - t_{{{\text{DR5}}}} } ) - D_{1} \overline{V}_{{{\text{b2}}}} \sqrt {t - t_{{{\text{DR5}}}} } }}{{C_{1} }},{ (}t_{{{\text{DR5}}}} { < }t{ < }t_{{{\text{FR}}}} )$$

where, $$A_{8}{\prime} = \frac{{\chi q_{{\text{s,b,i}}} H_{{\text{s}}} }}{L}$$, $$B_{8}{\prime} = \frac{14}{3}(T_{{\text{s}}} - T_{{\text{r}}} )\sqrt {\frac{{\lambda_{{{\text{cap}}}} \rho_{{{\text{cap}}}} c_{{{\text{cap}}}} }}{{\uppi }}} \overline{V}_{{_{5} }}$$, $$C_{1} = \frac{{\rho c(T_{{\text{s}}} - T_{{\text{r}}} )}}{{\rho_{{\text{o}}} \phi \Delta S_{{\text{o}}} }}$$, $$D_{1} = \frac{14}{3}(T_{{\text{s}}} - T_{{\text{r}}} )\sqrt {\frac{{\lambda_{{{\text{cap}}}} \rho_{{{\text{cap}}}} c_{{{\text{cap}}}} }}{{\uppi }}}$$.

### Mathematic model of the confinement stage

After the steam chamber moves to point F_R_ (see Fig. 11), the released heat rate per unit of horizontal well length in area 2 can be calculated by

90 $$q_{{{\text{inject}}}} = \frac{{\chi q_{{\text{s,b}}} H_{{\text{s}}} }}{L}$$

The rate of heat consumed per unit horizontal well length of steam chamber expansion is calculated by

91 $$q_{{{\text{in}}}} = \rho c(T_{{\text{s}}} - T_{{\text{r}}} )W\frac{{{\text{d}}y}}{{{\text{d}}t}},{ (}t > t_{{{\text{FR}}}} )$$

where, W is lateral drainage distance of SAGD, m.

According to Eq. (19), the cap rock heat loss during the confinement stage of steam chamber can be expressed as

92 $$q_{{{\text{cap}}}} = 2\int_{{t_{{\text{D}}} }}^{{t_{{{\text{FR}}}} }} {(T_{{\text{s}}} - T_{{\text{r}}} )\sqrt {\frac{{\lambda_{{{\text{cap}}}} \rho_{{{\text{cap}}}} c_{{{\text{cap}}}} }}{{{\uppi }(t - \tau )}}} } \overline{V}_{6} {\text{d}}\tau ,{ (}t > t_{{{\text{FR}}}} )$$

93 $$\overline{V}_{6} = \frac{{W - \frac{{w_{{\text{c}}} }}{2}}}{{t_{{{\text{FR}}}} - t_{{\text{D}}} }}$$

where, $\overline{V}_{6}$ is average movement speed of steam chamber from point D to point F_R_, m/d.

Combining Eqs. (14), (21), (48) and (90)−(93), we obtain

94 $$\frac{{\chi q_{{\text{s,b}}} H_{{\text{s}}} }}{L} = \rho c(T_{{\text{s}}} - T_{{\text{r}}} )W\frac{{{\text{d}}y}}{{{\text{d}}t}} + \frac{7}{3}\int_{{t_{{\text{D}}} }}^{{t_{{{\text{FR}}}} }} {(T_{{\text{s}}} - T_{{\text{r}}} )\sqrt {\frac{{\lambda_{{{\text{cap}}}} \rho_{{{\text{cap}}}} c_{{{\text{cap}}}} }}{{{\uppi }(t - \tau )}}} } \overline{V}_{6} {\text{d}}\tau ,{ (}t > t_{{{\text{FR}}}} )$$

Change the form of Eq. (94), we can get

95 $${\text{d}}y = \frac{{\frac{{\chi q_{{\text{s,b}}} H_{{\text{s}}} }}{L} - \frac{7}{3}\int_{{t_{{\text{D}}} }}^{{t_{{{\text{FR}}}} }} {(T_{{\text{s}}} - T_{{\text{r}}} )\sqrt {\frac{{\lambda_{{{\text{cap}}}} \rho_{{{\text{cap}}}} c_{{{\text{cap}}}} }}{{{\uppi }(t - \tau )}}} } \overline{V}_{6} {\text{d}}\tau }}{{\rho c(T_{{\text{s}}} - T_{{\text{r}}} )W}}{\text{d}}t,{ (}t > t_{{{\text{FR}}}} )$$

By integrating Eq. (95), the perpendicular front distance can be obtained by

96 $$y = \int_{{t_{{{\text{FR}}}} }}^{t} {\frac{{\frac{{\chi q_{{\text{s,b}}} H_{{\text{s}}} }}{L} - \frac{7}{3}\int_{{t_{{\text{D}}} }}^{{t_{{{\text{FR}}}} }} {(T_{{\text{s}}} - T_{{\text{r}}} )\sqrt {\frac{{\lambda_{{{\text{cap}}}} \rho_{{{\text{cap}}}} c_{{{\text{cap}}}} }}{{{\uppi }(t - \tau )}}} } \overline{V}_{6} {\text{d}}\tau }}{{\rho c(T_{{\text{s}}} - T_{{\text{r}}} )W}}{\text{d}}t} ,{ (}t > t_{{{\text{FR}}}} )$$

Based on the material balance principle, the oil production rate per unit of horizontal well length can be calculated by

97 $$q_{{\text{b}}} = \phi \Delta S_{{\text{o}}} \rho_{{\text{o}}} W\frac{{{\text{d}}y}}{{{\text{d}}t}},{ (}t > t_{{{\text{FR}}}} )$$

Substituting Eq. (96) into Eq. (97), we obtain

98 $$q_{{\text{b}}} = \phi \Delta S_{{\text{o}}} \rho_{{\text{o}}} \frac{{\frac{{\chi q_{{\text{s,b}}} H_{{\text{s}}} }}{L} - \frac{7}{3}\int_{{t_{{\text{D}}} }}^{{t_{{{\text{FR}}}} }} {(T_{{\text{s}}} - T_{{\text{r}}} )\sqrt {\frac{{\lambda_{{{\text{cap}}}} \rho_{{{\text{cap}}}} c_{{{\text{cap}}}} }}{{{\uppi }(t - \tau )}}} } \overline{V}_{6} {\text{d}}\tau }}{{\rho c(T_{{\text{s}}} - T_{{\text{r}}} )}},{ (}t > t_{{{\text{FR}}}} )$$

## Field application

### Example 1

There is a SAGD case from a heavy oil reservoir with an interlayer in the Junger Basin of China. The vertical depth of the steam injection well is 189.8 m, the vertical depth of the production well is 195.2 m, the perpendicular spacing between the injection and production wells is 5.4 m, and the horizontal section lengths of two wells are 377 m. The distances of the production well to the interlayer and the cap rock are 13.5 m and 31.8 m, respectively. Since 2016, the two horizontal wells have been put into production and injection for 2720 days, respectively.

**Figure 13:**
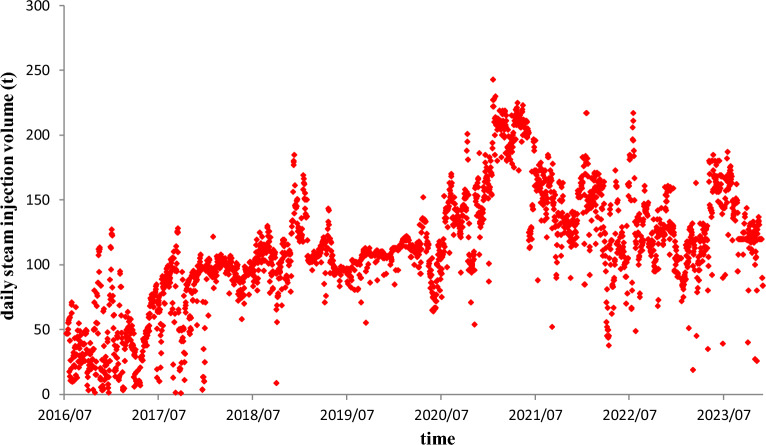
Relationship curve of steam injection rate with respect to time.

**Figure 14:**
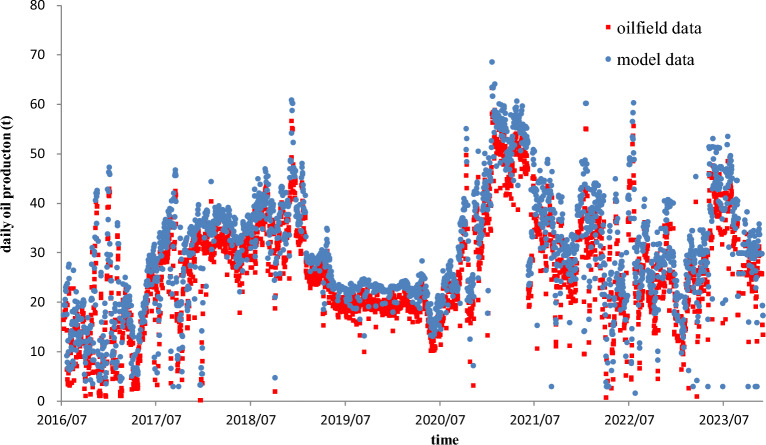
Matching curves of the model data with the oilfield data for the example 1 production well.

The physical property parameters of the heavy oil reservoir are shown in Table 1. Figure 13 shows the relationship curve of steam injection rate with respect to time. Various steam injection rate can be observed in the whole process of steam injection from the figure. Figure 14 shows the relationship curve of oil production rate with respect to time. Based on the physical property parameters and the history steam injection rate, we calculated the theoretical oil production rates with the elapse of production time by using the established SAGD model and then compared them with the real oil production rates, as shown in Fig. 14. It can be seen from the figure that the matching effect of the model data with the oilfield data is very well, which implies the established SAGD model is of reliability and utility.

**Table 1:** Values of physical property parameters of the example 1 reservoir.

*L*(m)	*ϕ*(j)	*H*(m)	*w*_c_(m)	*W*(m)	*S*_oi_(j)	*S*_or_(j)	*S*_wc_(j)	*h*(m)
377	0.311	31.8	40	50	0.749	0.30	0.251	13.5
*ρ*_r_ (kg/m^3^)	*ρ*_o_ (kg/m^3^)	*ρ*_w_ (kg/m^3^)	*c*_r_ J/(kg·°C)	*c*_o_ J/(kg·°C)	*c*_w_ J/(kg·°C)	*T*_s_ (°C)	*T*_r_ (°C)	*μ* (mPa s)
2500	963	1000	1000	3000	4200	210	19.6	14.5
*λ*_cap_ (J/(m day·°C))	*ρ*_cap_ (kg/m^3^)	*c*_cap_ (J/(kg·°C))	*λ*_layer_ (J/(m day·°C))	*H*_S_ (J/kg)	*χ*(j)	*α*(m^2^/s)	*k*(D)	*t*(day)
237,600	2500	1000	144,288	1900	0.85	5.9 × 10^–7^	2.16	2720

In addition, we also used the SAGD model to calculate the end time of each stage and the front position of steam chamber. The calculation results are shown in Table 2. The steam chamber rose to the interlayer on January 13, 2017, and then laterally expanded to the interlayer edge on June 22, 2018. After that, the steam chamber rose again to the cap rock on March 29, 2020, and then laterally expanded toward the lateral oil drainage boundary. As of December 2023, the steam chamber has not reached to the lateral drainage boundary. We also calculated the durations of different stages and periods, as listed in column 5 in Table 2. The durations of the first and second rising stages are 206 days and 636 days, respectively. The durations of the early and late periods of the first lateral expansion stage are 137 days and 388 days, respectively. The durations of the early and middle lateral expansion periods and the downward expansion period of area 1 are 468 days, 561 days and 324 days, respectively. The durations of the early and middle lateral expansion periods of area 2 are 468 days and 885 days, respectively. The calculation results can help us adequately understand the history development process and the current development status of steam chamber of the example SAGD.

**Table 2:** The end time and front positions of steam chamber in different stages.

Date	Steam injection rate (t/day)	Oil production rate (t/day)	Time (day)	Duration (day)	${|}{\text{x}}{|}$(m)	*z*(m)	Notes
2017/1/13	14.4	8.03	206	206	0.00	13.50	The first rising stage
2017/5/30	79	29.15	343	137	8.05	13.50	The early period of the first lateral expansion stage
2018/6/22	102.78	37.69	731	388	20.00	13.50	The late period of the first lateral expansion stage
2020/3/29	102	20.28	1367	636	20.00	31.80	The second rising stage
2021/6/30	177	44.24	1835	468	5.79	31.80	The early lateral expansion period of area 1
2023/1/12	85	14.34	2396	561	0.00	31.80	The middle lateral expansion period of area 1
2023/12/2	138	36.11	2720	324	0.00	14.76	The late downward expansion period of area 1
2021/6/30	177	44.24	1835	468	5.79	31.80	The early lateral expansion period of area 2
2023/12/2	138	36.11	2720	885	45.543	31.80	The middle lateral expansion period of area 2

Oil production rate is dominated by front position of steam chamber and steam injection rate. For example, at the end of the first rising stage, the vertical front distance of steam chamber is equal to 13.5 m, the steam injection rate is 14.4 t/day, and the corresponding oil production rate is 8.03 t/day. At the end of the early period of the first lateral expansion stage, the horizontal front distance of steam chamber is equal to 8.05 m, the steam injection rate is 79 t/day, and the corresponding oil production rate becomes 29.15 t/day. At the end of the second rising stage, the vertical and horizontal front distances of steam chamber become 20 m and 31.8 m, respectively, the steam injection rate is 102 t/day, and the corresponding oil production rate becomes 20.28 t/day.

### Example 2

There is another SAGD case from a heavy oil reservoir with an interlayer in the Junger Basin of China. The vertical depth of the steam injection well is 190.8 m, the vertical depth of the production well is 196.2 m, the perpendicular spacing between the injection and production wells is 5.4 m, and the horizontal section lengths of two wells are 346 m. The distances of the production well to the interlayer and the cap rock are 20 m and 38.8 m, respectively. Since 2019, the two horizontal wells have been put into production and injection for 1806 days, respectively.

**Figure 15:**
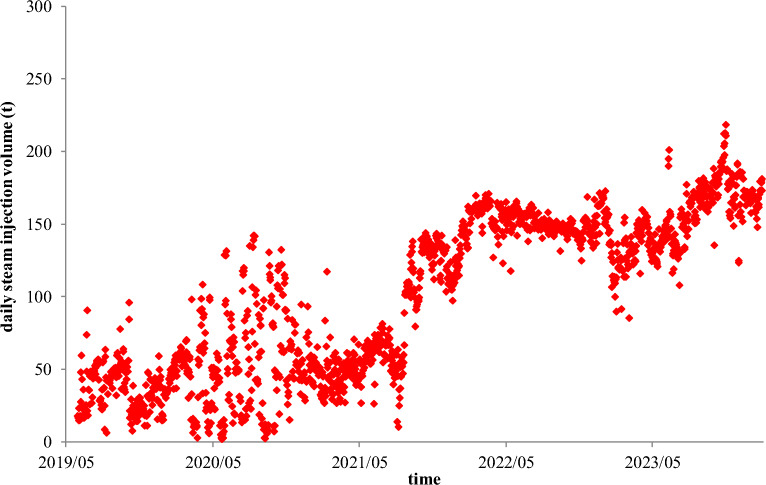
Relationship curve of steam injection rate with respect to time.

**Figure 16:**
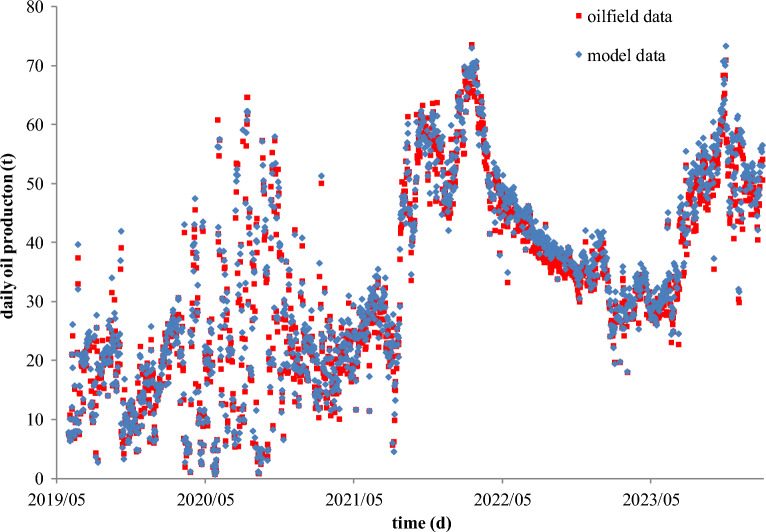
Matching curves of the model data with the oilfield data for the example 2 production well.

The physical property parameters of the heavy oil reservoir are shown in Table 3. Figure 15 shows the relationship curve of steam injection rate with respect to time. Figure 16 shows the relationship curve of oil production rate with respect to time. Based on the physical property parameters and the history steam injection rate, we again calculated the theoretical oil production rates with the elapse of production time by using the established SAGD model and then compared them with the real oil production rates, as shown in Fig. 16. From the figure, the matching effect between the production calculated by the model and the actual production of the oil field is very good, which once again proves that the SAGD model we established is reliable and practical.

**Table 3:** Values of physical property parameters of the example 2 reservoir.

*L*(m)	*ϕ*(j)	*H*(m)	*w*_c_(m)	*W*(m)	*S*_oi_(j)	*S*_or_(j)	*S*_wc_(j)	*h*(m)
346	0.303	38.8	52	60	0.761	0.31	0.239	24.4
*ρ*_r_ (kg/m^3^)	*ρ*_o_ (kg/m^3^)	*ρ*_w_ (kg/m^3^)	*c*_r_ J/(kg·°C)	*c*_o_ J/(kg·°C)	*c*_w_ J/(kg·°C)	*T*_s_ (°C)	*T*_r_ (°C)	*μ* (mPa·s)
2500	963	1000	1000	3000	4200	210	19.6	14.5
*λ*_cap_ (J/(m·d·°C))	*ρ*_cap_ (kg/m^3^)	*c*_cap_ (J/(kg·°C))	*λ*_layer_ (J/(m·d·°C))	*H*_S_ (J/kg)	*χ*(j)	*α*(m^2^/s)	*k*(D)	*t*(d)
237,600	2500	1000	144,288	1900	0.85	5.9 × 10^–7^	2.16	1806

In addition, we also used the SAGD model to calculate the end time of each stage and the front position of steam chamber. The calculation results are shown in Table 4. The steam chamber rose to the interlayer on July 8, 2020, and then laterally expanded to the interlayer edge on July 6, 2022. After that, the steam chamber rose again to the cap rock on January 13, 2024, and began to enter the second lateral expansion stage. As of January 2024, the steam chamber development is still in the second lateral expansion stage. We also calculated the durations of different stages and periods, as listed in column 5 in Table 4. The durations of the first and second rising stages are 405 days and 556 days, respectively. The durations of the early and late periods of the first lateral expansion stage are 476 days and 252 days, respectively.

**Table 4:** The end time and front positions of steam chamber in different stages.

Date	Steam injection volume (t/day)	Oil production (t/day)	Time (day)	Duration (day)	${|}{\text{x}}{|}$(m)	*z*(m)	Notes
2020/7/8	30.54	13.39	405	405	0.00	24.40	The first rising stage
2021/10/27	130.14	56.48	881	476	17.08	24.40	The early period of the first lateral expansion stage
2022/7/6	157.30	44.49	1133	252	26.00	24.40	The late period of the first lateral expansion stage
2024/1/13	166.88	50.52	1689	556	20.00	38.80	The second rising stage

Comparatively, the stage number of steam chamber development of the second example is lesser than that of the first example owing to the relatively shorter production time of the second example.

## Conclusions

In this study, multi-stage development process of steam chamber for SAGD production in a heavy oil reservoir with an interlayer was analyzed and the corresponding and multi-stage development model of steam chamber was established and solved. The following conclusions can be drawn:

1. The development process of steam chamber can be divided into 5 stages for a reservoir with an interlayer: the first rising stage (stage I), the first lateral expansion stage (stage II), the second rising stage (stage III), the second lateral expansion stage (stage IV) and the confinement stage (stage V). Stages III and IV are two special stages caused by the existence of the interlayer, and they do not appear for reservoirs without interlayers.
2. Particularly, stage II can be further divided into two periods: the early period and the late period. In stage IV, above the interlayer, the expansion process of steam chamber to the interior is different from that to the lateral drainage boundaries. Stage IV for the expansion to the interior can be further divided into three periods: the early and middle lateral expansion periods and the late downward expansion period. Stage IV for the expansion to the lateral drainage boundaries can also be further divided into three periods: the early, middle and late lateral expansion periods.
3. In stages I and II, the steam chamber is beneath the interlayer, and it can be assumed as an oval and an inverse triangle, respectively. In stage III, the steam chamber has already bypassed the interlayer and becomes two symmetric sub-chambers over the interlayer. The sub-chamber can also be hypothesized as an oval and the part of steam chamber beneath the interlayer can be deemed as a fixed inverse triangle. In stage IV, the sub-chamber can be considered as an inverse triangle. In stage V, the steam chamber can be treated as a polygon which is structured by a rectangle and an inverse triangle.
4. The established multi-stage development model is verified using field data applications and can be a good tool to calculate the front distance of steam chamber and oil production rate of SAGD in different development stages.

## Supplementary Information

Supplementary Information.

## Data Availability

Data will be made available on request, pleases contact the first author.

## Abbreviations

*q*: Oil production rate per unit of horizontal well length, kg/(m day)
*a*: Major radius of oval chamber, m
*S*_oi_: Initial oil saturation, (j)
*S*_or_: Residual oil saturation, (j)
*t*: Time, day
*q*_inject_: Heat rate per unit of horizontal well length, J/(m day)
*q*_s_: Steam injection rate, kg/day
*H*_s_: Latent heat of steam, J/kg
*L*: Horizontal well length, m
*q*_r_: Heat absorption rate of rock per unit of horizontal well length, J/(m day)
*q*_o_: Heat absorption rate of heavy oil per unit of horizontal well length, J/(m day)
*q*_wc_: Heat absorption rate of irreducible water per unit of horizontal well length, J/(m day)
*S*_wc_: Irreducible water saturation, %
*c*_r_: Specific heat of rock, J/(kg·°C)
*c*_o_: Specific heat of oil, J/(kg·°C)
*c*_*w*_: Specific heat of water, J/(kg·°C)
*T*_s_: Steam chamber temperature, °C
*T*_r_: Initial reservoir temperature, °C
*h*_1_: Rising height of oval steam chamber, m
*v*: Rising speed of steam chamber, m/day
*q*_in_: Rate of heat absorption inside the steam chamber per unit horizontal well length, J/(m day)
*q*_loss_: Rate of heat loss per unit horizontal well length, J/(m day)
*Q*_in_: Heat consumed per unit horizontal well length of steam chamber expansion, J/m
*h*: Distance from the interlayer to the production well, m
*q*_layer_: Rate of heat loss at the interlayer per unit horizontal well length, J/(m day)
*q*_side_: Rate of heat loss at the side interface of steam chamber per unit horizontal well length; J/(m day)
*q*_cap_: Rate of heat loss at the cap rock per unit horizontal well length, J/(m^2^ day)
*c*_cap_: Cap rock heat capacity, J/(kg·°C)
*c*_layer_: Interlayer heat capacity, J/(kg·°C)
*t*_c_: Time for the front position to reach point C, day
erfc: Error function
*Q*_inject_: Latent heat released by steam per unit horizontal well length, J/m
*t*_CR4_: Time of steam chamber reaching to point C_R4_, day
$\overline{q}_{{\text{s}}}$: Average rate of steam injection from *t*_CR4_ to *t*, kg/day
*t*_ER_: Time of steam chamber reaching to point E_R_, day
*q*_s,__*i*_: Steam injection rate on the *i*^th^ day, kg/day
$\overline{V}$: Average movement speed of the steam chamber during the late period of the first lateral expansion stage, m/day
*Q*_loss_: Heat loss per unit horizontal well length of steam chamber expansion, J/m
$\overline{V}_{{1}}$: Average movement speed of steam chamber from *t*_C_ to *t*_CR4_, m/day
*x*_CCR4_: Length of line CC_R4_, m
$\overline{V}_{2}$: Average movement speed of sub-chamber from *t*_C_ to *t*_ER_, m/day
*w*_c_: Interlayer width, m
*a*_2_: Major radius of oval chamber in the second rising stage, m
*h*_2_: Rising height of oval sub-chamber, m
*H*: Perpendicular distance from the production well to the cap rock, m
*t*_D_: Time of steam chamber reaching to point D, day
*t*_DL3_: Time of steam chamber reaching to point D_L3_, day
*t*_M_: Time of steam chamber reaching to point M, day
$\overline{V}_{{\text{a}}}$: The average movement speed of sub-chamber from point D_L3_ to point D_M_
$\overline{V}_{{3}}$: Average movement speed of sub-chamber from point D to point D_L3_, m/day
*x*_DDL3_: Length of line DD_L3_, m
*k*: Effective permeability of oil flow, m^2^
*m*: A coefficient constant
*v*_so_: Kinematic viscosity of oil at the temperature inside steam chamber, m^2^/day
*q*_a_: Oil production rate per unit of horizontal well in area 1, m^3^/(m day)
*q*_s,a_: Steam flow rate in area 1, kg/day
$\overline{V}_{{4}}$: Average speed of sub-chamber from point D to point M, m/day
*y*: Perpendicular front distance, m
$\overline{q}_{{\text{s,b}}}$: Average steam flow rate in area 2, kg/day
*q*_s,b,__*i*_: Steam flow rate on the *i*th day in area 2, kg/day
$\overline{V}_{{\text{b}}}$: Average movement speed of sub-chamber from point D_M_ to point D_R5_, m/day
$\overline{V}_{{{\text{b2}}}}$: Average movement speed of sub-chamber from point D_R5_ to point F_R_, m/day
*t*_FR_: Time of steam chamber reaching to point F_R_, day
$\overline{V}_{5}$: Average speed of sub-chamber from point D to point D_R5_, m/day
*x*_DDR5_: Length of line DD_R5_, m
W: Lateral drainage distance of SAGD, m
$\overline{V}_{6}$: Average movement speed of steam chamber from point D to point F_R_, m/day
*α*: Thermal diffusivity of reservoir rock, m^2^/day
Г( ): Gamma function
*η*: Experience coefficient, generally 0.7
*λ*_cap_: Thermal conduction coefficient of cap rock, J/(m day·°C)
*λ*_layer_: Thermal conduction coefficient of the interlayer, J/(m day·°C)
*ρ*_o_: Oil density, kg/m^3^
*ρ*_r_: Rock density, kg/m^3^
*ρ*_*w*_: Water density, kg/m^3^
*ρ*_layer_: Interlayer density, kg/m^3^
*ρ*_cap_: Cap rock density, kg/m^3^
*τ*: Integral variable respect to time, day
*ϕ*: Porosity, (j)
*χ*: Steam quality, (j)

## References

[1] Gomaa, S., Salem, K. G. & El-hoshoudy, A. N. Recovery of heavy oil and extra heavy oil; Current status, new trends, and enhancement techniques. *Petroleum*10.1016/j.petlm.2023.10.001 (2023).

[2] Wang, L. *et al.* A comprehensive investigation of SAGD steam chamber in dual horizontal well pairs: Expansion angel and connection characteristics. *J. Pet. Sci. Eng.* **217**, 110888. 10.1016/j.petrol.2022.110888 (2022).

[3] Butler, R. M. & Stephens, D. J. The gravity drainage of steam-heated heavy oil to parallel horizontal wells. *J. Can. Pet. Technol.* **20**(2), 90–96. 10.2118/81-02-07 (1981).

[4] Huang, S., Yang, L., Xia, Y., Du, M. & Yang, Y. An experimental and numerical study of a steam chamber and production characteristics of SAGD considering multiple barrier layers. *J. Pet. Sci. Eng.* **180**, 716–726. 10.1016/j.petrol.2019.05.062 (2019).

[5] Guo, Y., Liu, H., Feng, Y., Dong, X. & Zheng, W. A new SAGD comprehensive multi-stage model for oil production using a concave parabola geometry. *J. Pet. Sci. Eng.* **208**, 109321. 10.1016/j.petrol.2021.109321 (2022).

[6] Butler, R. M. Steam-assisted gravity drainage: Concept, development, performance and future. *J. Can. Pet. Technol.* **33**(2), 44–50. 10.2118/94-02-05 (1994).

[7] Sedaee Sola, B. & Rashidi, F. Application of the SAGD to an Iranian Carbonate Heavy-Oil Reservoir. *Proc. SPE West. Reg. AAPG Pac. Sect. GSA Cordilleran Sect. Jt. Meet.*10.2523/100533-MS (2006).

[8] Shaolei, W., Linsong, C., Wenjun, H., Shijun, H. & Shuai, L. Prediction for steam chamber development and production performance in SAGD process. *J. Nat. Gas Sci. Eng.* **19**, 303–310. 10.1016/j.jngse.2014.05.021 (2014).

[9] Cui, G., Liu, T., Xie, J., Rong, G. & Yang, L. A review of SAGD technology development and its possible application potential on thin-layer super-heavy oil reservoirs. *Geosci. Front.* **13**(4), 101382. 10.1016/j.gsf.2022.101382 (2022).

[10] Edmunds, N. R., Kovalsky, J. A., Gittins, S. D. & Pennacchioli, E. D. Review of phase a steam-assisted gravity drainage test. *SPE Reserv. Eng.* **9**(2), 119–124. 10.2118/21529-PA (1994).

[11] Zargar, Z. & Ali, S. M. F. Analytical treatment of steam-assisted gravity drainage: Old and new. *SPE J.* **23**(01), 117–127. 10.2118/180748-PA (2017).

[12] Zargar, Z. & Ali, S. M. F. Analytical modelling of steam chamber rise stage of Steam-Assisted Gravity Drainage (SAGD) process. *Fuel* **233**, 732–742. 10.1016/j.fuel.2018.06.106 (2018).

[13] Shi, L. *et al.* Analytical modeling of oil production rate during the entire steam-assisted gravity drainage process in heavy oil reservoirs. *J. Pet. Sci. Eng.* **175**, 190–199. 10.1016/j.petrol.2018.12.040 (2019).

[14] Azad, A. & Chalaturnyk, R. J. An improved SAGD analytical simulator: Circular steam chamber geometry. *J. Pet. Sci. Eng.* **82–83**, 27–37. 10.1016/j.petrol.2012.01.003 (2012).

[15] Nie, R.-S., Wang, Y.-M., Kang, Y.-L. & Jia, Y.-L. A steam rising model of steam-assisted gravity drainage production for heavy oil reservoirs. *Energy Explor. Exploit.* **38**(4), 801–818. 10.1177/0144598719897178 (2019).

[16] Zargar, Z., Farouq Ali, S. M. Comprehensive multi-stage analytical treatment of steam-assisted gravity drainage SAGD. In *Presented at the 2018 SPE Annual Technical Conference and Exhibition held in Dallas, Texas, 24–26 September 2018*. 10.2118/191533-MS.

[17] Zargar, Z., Razavi, S. M. & Ali, S. M. F. Analytical model of steam-assisted gravity drainage (SAGD) process in relation to constant injection rate. *Fuel* **265**, 116772. 10.1016/j.fuel.2019.116772 (2020).

[18] Zhang, Q. *et al.* A new comprehensive model to estimate the steam chamber expansion and recovery performance of entire SAGD process. *J. Pet. Sci. Eng.* **185**, 106629. 10.1016/j.petrol.2019.106629 (2020).

[19] Butler, R. M., Mcnab, G. S. & Lo, H. Y. Theoretical studies on the gravity drainage of heavy oil during in-situ steam heating. *Can. J. Chem. Eng.* **59**(4), 455–460. 10.1002/cjce.5450590407 (1981).

[20] Reis, J. C. A steam-assisted gravity drainage model for tar sands: linear geometry. *J. Can. Pet. Technol*. **31**(10), PETSOC-92-10-01. 10.2118/92-10-01 (1992).

[21] Reis, J. C. A steam assisted gravity drainage model for tar sands: radial geometry. *J. Can. Pet. Technol.* **32**(08), PETSOC-93-08-05. 10.2118/93-08-05 (1993).

[22] Akin, S. Mathematical modeling of steam-assisted gravity drainage. *Comput. Geosci.* **32**(2), 240–246. 10.1016/j.cageo.2005.06.007 (2006).

[23] Azad, A., Chalaturnyk, R. J. Geomechanical coupling simulation in SAGD process: A linear geometry model. In *Presented at the 3rd Canada-US (CANUS) Rock Mechanics Symposium and 20th Canadian Rock Mechanics Symposium, Rock Engineering in Difficult Conditions, Toronto, Canada, May, 2009*. https://api.semanticscholar.org/CorpusID:189759599.

[24] Azad, A. & Chalaturnyk, R. J. A mathematical improvement to SAGD using geomechanical modelling. *J. Can. Pet. Technol.* **49**(10), 53–64. 10.2118/141303-PA (2010).

[25] Igbokwe, L. C., Obumse, C. M. & Hossain, M. E. New SAGD model for oil production using a concave parabola steam chamber geometry. *J. Pet. Sci. Eng.* **175**, 971–984. 10.1016/j.petrol.2018.12.052 (2019).

[26] Sabeti, M., Rahimbakhsh, A. & Mohammadi, A. H. Using exponential geometry for estimating oil production in the SAGD process. *J. Pet. Sci. Eng.* **138**, 113–121. 10.1016/j.petrol.2015.11.014 (2016).

[27] Shin, H., Choe, J. Shale Barrier effects on the SAGD performance. In *Presented at the 2009 SPE/EAGE Reservoir Characterization and Simulation Conference, Abu Dhabi, UAE, 19–21 October, 2009*. 10.2118/125211-MS.

[28] Dang, T. Q. C., Chen, Z. & Nguyen, T. B. N. Numerical simulation of SAGD recovery process in presence of shale barriers, thief zones, and fracture system. *Pet. Sci. Technol.* **31**(14), 1454–1470. 10.1080/10916466.2010.545792 (2013).

[29] Wang, Q. Q., Lin, B. T., Jin, Y. Numerical simulation on SAGD recovery in terrestrial heavy oil reservoirs considering influences of mudstone stringers. In *Presented at the 52nd U.S. Rock Mechanics/Geomechanics Symposium, Seattle, Washington, June 2018*. http://refhub.elsevier.com/S0920-4105(19)30516-9/sref34.

[30] Xiong, H. *et al.* Influence of pressure difference between reservoir and production well on steam-chamber propagation and reservoir-production performance. *SPE J.* **24**(02), 452–476. 10.2118/190107-PA (2019).

[31] Kumar, A. & Hassanzadeh, H. Impact of shale barriers on performance of SAGD and ES-SAGD—A review. *Fuel* **289**, 119850. 10.1016/j.fuel.2020.119850 (2021).

[32] Ipek, G., Frauenfeld, T., Yuan, J. Y. Numerical study of shale issues in SAGD. In *Presented at the Proceedings of the Canadian International Petroleum Conference/SPE Gas Technology Symposium 2008 Joint Conference, Calgary, Alberta, Canada, 17–19 June, 2008*. 10.2118/2008-150.

[33] Fatemi, S. M. The Effect of geometrical properties of reservoir shale barriers on the performance of steam-assisted gravity drainage (SAGD). *Energy Sources Part A Recov. Util. Environ. Eff.* **34**(23), 2178–2191. 10.1080/15567036.2010.497796 (2012).

[34] Huang, S., Yang, L., Xia, Y., Du, M. & Yang, Y. An experimental and numerical study of a steam chamber and production characteristics of SAGD considering multiple barrier layers. *J. Pet. Sci. Eng.* **180**, 716–726. 10.1016/j.fuel.2016.06.104 (2019).

[35] Wei, S. *et al.* Experimental study on the effect of different distributed interlayer on SAGD performance. *J. Pet. Sci. Eng.* **209**, 109827. 10.1016/j.petrol.2021.109827 (2022).

[36] Zargar, Z. & Ali, S. M. F. Effect of confinement and well interference on SAGD performance: An analytical assessment. *SPE J.* **24**(04), 1595–1612. 10.2118/189715-PA (2019).

[37] Carslaw, H. S. & Jaeger, J. C. Conduction of heat in solids, second edition. *J. Eng. Mater. Technol. Trans. ASME* **108**(4), 378. 10.1115/1.3225900 (1986).

