# Supplementary material for: Multi-stage development process and model of steam chamber for SAGD production in a heavy oil reservoir with an interlayer
Source: Sci Rep. 2024 Apr 30;14:9959. doi: 10.1038/s41598-024-60747-7 (PMC11582660; doi:10.1038/s41598-024-60747-7)
Supplement: Supplementary file 1 — Supplementary Information. [file 41598_2024_60747_MOESM1_ESM.docx]

## Appendices

## Appendix A: Deduction of the front distance of steam chamber in the early period of the first lateral expansion stage

Let, $A_{2}=\frac{\chi q_{\text{s}}H_{\text{s}}}{L}$, $B_{2}=\frac{7}{3}(T_{\text{s}}-T_{\text{r}})\sqrt{\frac{\lambda_{\text{layer}}\rho_{\text{layer}}c_{\text{layer}}}{\text{π}}}$, and $C=\rho c(T_{\text{s}}-T_{\text{r}})\frac{h}{2}$, and substitute these equations into equation (22):

$$A_{2}=C\frac{\text{d}x}{\text{d}t}+B_{2}\int_{t_{\text{C}}}^{t} \sqrt{\frac{1}{(t-\tau)}}\frac{\text{d}x}{\text{d}\tau}\text{d}\tau,\text{ (}t_{\text{C}}\text{<}t<t_{\text{CR4}})$$

The Laplace transform based on *t* is introduced:

$$L[x(t)]=X(s)=\int_{0}^{\infty} x(t)\text{e}^{-st}\text{d}t$$

Where *s* is Laplace variable; X is front distance of steam chamber in the Laplace space.

Take the Laplace transform to equation (A-1):

$$\frac{A_{2}}{s}-B_{2}\cdot\Gamma(0.5)\sqrt{s}\cdot X=C\cdot s\cdot X$$

Where, Г( ) is the gamma function.

The solution of equation (A-3) in Laplace space,

$$X=\frac{A_{2}}{sC}\frac{1}{\sqrt{s}(\sqrt{s}+\frac{B_{2}\Gamma(0.5)}{C})}$$

Using inverse Laplace transformation to equation (A-4), the front distance of steam chamber in the early period of the first lateral expansion stage can be obtained by

$$x=\int_{t_{\text{C}}}^{t} \frac{A_{2}}{C}\text{e}^{[\frac{B_{2}}{C}\Gamma(0.5)]^{2}\tau}\text{erfc}[\frac{B_{2}}{C}\Gamma(0.5)\sqrt{\tau}]\text{d}\tau,\text{ (}t_{\text{C}}\text{<}t<t_{\text{CR4}})$$

## Appendix B: Deduction of the front distance of steam chamber and oil production rate in the late period of the first lateral expansion stage

Let $A_{3}=\frac{\chi\overline{q}_{\text{s}}H_{\text{s}}}{L}$, $B_{3}=\frac{28}{9}(T_{\text{s}}-T_{\text{r}})\sqrt{\frac{\lambda_{\text{layer}}\rho_{\text{layer}}c_{\text{layer}}}{\text{π}}}\overline{V}_{1}$, $C=\rho c(T_{\text{s}}-T_{\text{r}})h$,

$D=\frac{28}{9}(T_{\text{s}}-T_{\text{r}})\sqrt{\frac{\lambda_{\text{layer}}\rho_{\text{layer}}c_{\text{layer}}}{\text{π}}}$ and substitute these equations into equation (34):

$$A_{3}-B_{3}[\frac{(t-t_{\text{C}})^{\frac{3}{2}}-(t_{\text{CR4}}-t_{\text{C}})^{\frac{3}{2}}}{t-t_{\text{CR4}}}-\sqrt{t-t_{\text{CR4}}}]=C\overline{V}+D\overline{V}\sqrt{t-t_{\text{CR4}}},\text{ (}t_{\text{CR4}}<t<t_{\text{ER}})$$

Change the form of equation (B-1), we can obtain

$$\overline{V}=\frac{A_{3}-B_{3}\{[(t-t_{\text{C}})^{\frac{3}{2}}-(t_{\text{CR4}}-t_{\text{C}})^{\frac{3}{2}}]/(t-t_{\text{CR4}})-\sqrt{t-t_{\text{CR4}}}\}}{C+D\sqrt{t-t_{\text{CR4}}}},\text{ (}t_{\text{CR4}}<t<t_{\text{ER}})$$

According to equation (B-2), the front distance of steam chamber in the late period of the first lateral expansion stage can be obtained by

$$x=\frac{A_{3}-B_{3}\{[(t-t_{\text{C}})^{\frac{3}{2}}-(t_{\text{CR4}}-t_{\text{C}})^{\frac{3}{2}}]/(t-t_{\text{CR4}})-\sqrt{t-t_{\text{CR4}}}\}}{C+D\sqrt{t-t_{\text{CR4}}}}(t-t_{\text{CR4}}),\text{ (}t_{\text{CR4}}<t<t_{\text{ER}})$$

Based on the material balance principle, the oil production rate per unit of horizontal well length can be calculated by

The rate of heat consumed per unit horizontal well length of steam chamber expansion is calculated by

$$q_{\text{in}}==\rho c(T_{\text{s}}-T_{\text{r}})h\frac{\text{d}x}{\text{d}t},\text{ (}t_{\text{CR4}}<t<t_{\text{ER}})$$

Combining equations (14), (21), (18) (31) (B-4) and (B-5), we obtain

Let, $A_{3}^{'}=\frac{\chi q_{\text{s,i}}H_{\text{s}}}{L}$; $B_{3}^{'}=\frac{14}{3}(T_{\text{s}}-T_{\text{r}})\sqrt{\frac{\lambda_{\text{layer}}\rho_{\text{layer}}c_{\text{layer}}}{\text{π}}}\overline{V}_{1}$; ;

$$D_{1}=\frac{14}{3}(T_{\text{s}}-T_{\text{r}})\sqrt{\frac{\lambda_{\text{layer}}\rho_{\text{layer}}c_{\text{layer}}}{\text{π}}}$$

Equation (B-6) is reduced to

$$A_{3}^{'}-B_{3}^{'}(\sqrt{t-t_{\text{C}}}-\sqrt{t-t_{\text{CR4}}})=C_{1}q+D_{1}\overline{V}\sqrt{t-t_{\text{CR4}}},\text{ (}t_{\text{CR4}}<t<t_{\text{ER}})$$

By changing the form of equation (B-7), the oil production rate per unit of horizontal well length can be calculated by

## Appendix C: Deduction of the front distance of sub-chamber in the early lateral expansion period of area 1

Let, $A_{5}=\frac{\chi q_{\text{s}}H_{\text{s}}}{L}$, $B_{5}=\frac{14}{3}(T_{\text{s}}-T_{\text{r}})\sqrt{\frac{\lambda_{\text{layer}}\rho_{\text{layer}}c_{\text{layer}}}{\text{π}}}$, and $C=\rho c(T_{\text{s}}-T_{\text{r}})\frac{h}{2}$, and substitute these equations into equation (50):

$$A_{5}=C\frac{\text{d}x}{\text{d}t}+B_{5}\int_{t_{\text{D}}}^{t} \sqrt{\frac{1}{(t-\tau)}}\frac{\text{d}x}{\text{d}\tau}\text{d}\tau,\text{ (}t_{\text{D}}<t<t_{\text{DL3}})$$

By combining equations (A-1) − (A-4) in Appendix A, the front distance of sub-chamber in the early period of the second lateral expansion stage can be obtained by

$$x=\int_{t_{\text{D}}}^{t} \frac{A_{5}}{C}\text{e}^{[\frac{B_{5}}{C}\Gamma(0.5)]^{2}\tau}\text{erfc}[\frac{B_{2}}{C}\Gamma(0.5)\sqrt{\tau}]\text{d}\tau$$

## Appendix D: Deduction of the front distance of sub-chamber and oil production rate in the middle lateral expansion period of area 1

Let $A_{6}=\frac{\chi\overline{q}_{\text{s}}H_{\text{s}}}{2L}$, $B_{6}=\frac{28}{9}(T_{\text{s}}-T_{\text{r}})\sqrt{\frac{\lambda_{\text{cap}}\rho_{\text{cap}}c_{\text{cap}}}{\text{π}}}\overline{V}_{3}$, $C=\rho c(T_{\text{s}}-T_{\text{r}})(H-h)$,

$D=\frac{28}{9}(T_{\text{s}}-T_{\text{r}})\sqrt{\frac{\lambda_{\text{cap}}\rho_{\text{cap}}c_{\text{cap}}}{\text{π}}}$, and substitute these equations into equation (61):

$$A_{6}-B_{6}[\frac{(t-t_{\text{D}})^{\frac{3}{2}}-(t_{\text{DL3}}-t_{\text{D}})^{\frac{3}{2}}}{t-t_{\text{DL3}}}-\sqrt{t-t_{\text{DL3}}}]=C\overline{V}_{\text{a}}+D\overline{V}_{\text{a}}\sqrt{t-t_{\text{DL3}}},\text{ (}t_{\text{DL3}}<t<t_{\text{M}})$$

Changing the form of equation (D-1), we can get

$$\overline{V}_{\text{a}}=\frac{A_{6}-B_{6}[\frac{(t-t_{\text{D}})^{\frac{3}{2}}-(t_{\text{DL3}}-t_{\text{D}})^{\frac{3}{2}}}{t-t_{\text{DL3}}}-\sqrt{t-t_{\text{DL3}}}]}{C+D\sqrt{t-t_{\text{DL3}}}},\text{ (}t_{\text{DL3}}<t<t_{\text{M}})$$

According to equation (D-2), the front distance of sub-chamber in the middle lateral expansion period of area 1 can be obtained by

$$x=\frac{A_{6}-B_{6}[\frac{(t-t_{\text{D}})^{\frac{3}{2}}-(t_{\text{DL3}}-t_{\text{D}})^{\frac{3}{2}}}{t}-\sqrt{t-t_{\text{DL3}}}]}{C+D\sqrt{t-t_{\text{DL3}}}}(t-t_{\text{DL3}}),\text{ (}t_{\text{DL3}}<t<t_{\text{M}})$$

Based on the material balance principle, the oil production rate per unit of horizontal well length can be calculated by

The rate of heat consumed per unit horizontal well length of steam chamber expansion is calculated by

$$q_{\text{in}}=\rho c(T_{\text{s}}-T_{\text{r}})(H-h)\frac{\text{d}x}{\text{d}t},\text{ (}t_{\text{DL3}}<t<t_{\text{M}})$$

Combining equations (14), (18), (21), (57), (60), (D-4) and (D-5), we obtain

Let, $A_{6}^{'}=\frac{\chi q_{\text{s,i}}H_{\text{s}}}{L}$; $B_{6}^{'}=\frac{14}{3}(T_{\text{s}}-T_{\text{r}})\sqrt{\frac{\lambda_{\text{layer}}\rho_{\text{layer}}c_{\text{layer}}}{\text{π}}}\overline{V}_{3}$; ;

$$D_{1}=\frac{14}{3}(T_{\text{s}}-T_{\text{r}})\sqrt{\frac{\lambda_{\text{cap}}\rho_{\text{cap}}c_{\text{cap}}}{\text{π}}}$$

Equation (D-6) is reduced to

By changing the form of equation (D-7), the oil production rate per unit of horizontal well length can be calculated by

## Appendix E: Deduction of the front distance of sub-chamber and oil production rate in the middle lateral expansion period of area 2

Let $A_{7}=\frac{\chi\overline{q}_{\text{s,b}}H_{\text{s}}}{L}$, $B_{7}=\frac{28}{9}(T_{\text{s}}-T_{\text{r}})\sqrt{\frac{\lambda_{\text{cap}}\rho_{\text{cap}}c_{\text{cap}}}{\text{π}}}\overline{V}_{3}$, $C=\rho c(T_{\text{s}}-T_{\text{r}})(H-h)$,

$D=\frac{28}{9}(T_{\text{s}}-T_{\text{r}})\sqrt{\frac{\lambda_{\text{cap}}\rho_{\text{cap}}c_{\text{cap}}}{\text{π}}}$, and substitute these equations into equation (78):

$$A_{7}-B_{7}[\frac{(t-t_{\text{D}})^{\frac{3}{2}}-(t_{\text{DM}}-t_{\text{D}})^{\frac{3}{2}}}{t-t_{\text{DM}}}-\sqrt{t-t_{\text{DM}}}]=C\overline{V}_{\text{b}}+D\overline{V}_{\text{b}}\sqrt{t-t_{\text{DM}}},\text{ (}t_{\text{DM}}\text{<}t\text{<}t_{\text{DR5}})$$

Changing the form of equation (E-1), we can get

$$\overline{V}_{\text{a}}=\frac{A_{7}-B_{7}[\frac{(t-t_{\text{D}})^{\frac{3}{2}}-(t_{\text{DM}}-t_{\text{D}})^{\frac{3}{2}}}{t-t_{\text{DM}}}-\sqrt{t-t_{\text{DM}}}]}{C+D\sqrt{t-t_{\text{DM}}}},\text{ (}t_{\text{DM}}\text{<}t\text{<}t_{\text{DR5}})$$

According to equation (E-2), the front distance of sub-chamber in the middle lateral expansion period of area 2 can be obtained by

$$x=\frac{A_{7}-B_{7}[\frac{(t-t_{\text{D}})^{\frac{3}{2}}-(t_{\text{DM}}-t_{\text{D}})^{\frac{3}{2}}}{t-t_{\text{DM}}}-\sqrt{t-t_{\text{DM}}}]}{C+D\sqrt{t-t_{\text{DM}}}}(t-t_{\text{DM}}),\text{ (}t_{\text{DM}}\text{<}t\text{<}t_{\text{DR5}})$$

Based on the material balance principle, the oil production rate per unit of horizontal well length can be calculated by

The rate of heat consumed per unit horizontal well length of steam chamber expansion is computed by

$$q_{\text{in}}=\rho c(T_{\text{s}}-T_{\text{r}})(H-h)\frac{\text{d}x}{\text{d}t},\text{ (}t_{\text{DM}}\text{<}t\text{<}t_{\text{DR5}})$$

Combining equations (21), (29), (48), (76), (77), (E-4) and (E-5), we obtain

Let, $A_{7}^{'}=\frac{\chi q_{\text{s,b,i}}H_{\text{s}}}{L}$; $B_{7}^{'}=\frac{14}{3}(T_{\text{s}}-T_{\text{r}})\sqrt{\frac{\lambda_{\text{cap}}\rho_{\text{cap}}c_{\text{cap}}}{\text{π}}}\overline{V}_{_{3}}$; ;

$$D_{1}=\frac{14}{3}(T_{\text{s}}-T_{\text{r}})\sqrt{\frac{\lambda_{\text{layer}}\rho_{\text{layer}}c_{\text{layer}}}{\text{π}}}$$

Equation (E-6) is reduced to

By changing the form of equation (E-7), the oil production rate per unit of horizontal well length can be calculated by

## Appendix F: Deduction of the front distance of sub-chamber and oil production rate in the late lateral expansion period of area 2

Let $A_{7}=\frac{\chi\overline{q}_{\text{s,b}}H_{\text{s}}}{L}$, $B_{7}=\frac{28}{9}(T_{\text{s}}-T_{\text{r}})\sqrt{\frac{\lambda_{\text{cap}}\rho_{\text{cap}}c_{\text{cap}}}{\text{π}}}\overline{V}_{3}$, $C=\rho c(T_{\text{s}}-T_{\text{r}})H$,

$D=\frac{28}{9}(T_{\text{s}}-T_{\text{r}})\sqrt{\frac{\lambda_{\text{cap}}\rho_{\text{cap}}c_{\text{cap}}}{\text{π}}}$, and substitute these equations into equation (87):

$$A_{8}-B_{8}[\frac{(t-t_{\text{D}})^{\frac{3}{2}}-(t_{\text{DR5}}-t_{\text{D}})^{\frac{3}{2}}}{t-t_{\text{DR5}}}-\sqrt{t-t_{\text{DR5}}}]=C\overline{V}_{b2}+D\overline{V}_{b2}\sqrt{t-t_{\text{DR5}}},\text{ (}t_{\text{DR5}}\text{<}t\text{<}t_{\text{FR}})$$

Changing the form of equation (F-1), we can get

$$\overline{V}_{b2}=\frac{A_{8}-B_{8}[\frac{(t-t_{\text{D}})^{\frac{3}{2}}-(t_{\text{DR5}}-t_{\text{D}})^{\frac{3}{2}}}{t-t_{\text{DR5}}}-\sqrt{t-t_{\text{DR5}}}]}{C+D\sqrt{t-t_{\text{DR5}}}},\text{ (}t_{\text{DR5}}\text{<}t\text{<}t_{\text{FR}})$$

According to equation (F-2), the front distance of sub-chamber in the late lateral expansion period of area 2 can be obtained by

$$x=\frac{A_{8}-B_{8}[\frac{(t-t_{\text{D}})^{\frac{3}{2}}-(t_{\text{DR5}}-t_{\text{D}})^{\frac{3}{2}}}{t-t_{\text{DR5}}}-\sqrt{t-t_{\text{DR5}}}]}{C+D\sqrt{t-t_{\text{DR5}}}}(t-t_{\text{DR5}}),\text{ (}t_{\text{DR5}}\text{<}t\text{<}t_{\text{FR}})$$

Based on the material balance principle, the oil production rate per unit of horizontal well length can be calculated by

The rate of heat consumed per unit horizontal well length of steam chamber expansion is calculated by

$$q_{\text{in}}=\rho c(T_{\text{s}}-T_{\text{r}})H\frac{\text{d}x}{\text{d}t},\text{ (}t_{\text{DR5}}\text{<}t\text{<}t_{\text{FR}})$$

Combining equations (21), (29), (48), (84), (86), (F-4) and (F-5), we obtain

Let, $A_{8}^{'}=\frac{\chi q_{\text{s,b,i}}H_{\text{s}}}{L}$; $B_{8}^{'}=\frac{14}{3}(T_{\text{s}}-T_{\text{r}})\sqrt{\frac{\lambda_{\text{cap}}\rho_{\text{cap}}c_{\text{cap}}}{\text{π}}}\overline{V}_{_{5}}$; ;

$$D_{1}=\frac{14}{3}(T_{\text{s}}-T_{\text{r}})\sqrt{\frac{\lambda_{\text{cap}}\rho_{\text{cap}}c_{\text{cap}}}{\text{π}}}$$

Equation (F-6) is reduced to

By changing the form of equation (F-7), the oil production rate per unit of horizontal well length can be calculated by
